# Production optimization and *in vitro* evaluation of the biofunctional potential of folate from *Lactiplantibacillus plantarum*-MGKMVIT11

**DOI:** 10.3389/fmicb.2026.1811272

**Published:** 2026-05-15

**Authors:** G. Megala, M. Kavitha

**Affiliations:** School of Bio Sciences and Technology, Vellore Institute of Technology, Vellore, Tamil Nadu, India

**Keywords:** anti-inflammatory activity, antioxidant, drug interaction analysis, folate, *Lactiplantibacillus plantarum* MGKMVIT11, optimization, probiotic potential

## Abstract

**Introduction:**

Folate-producing probiotic bacteria have gained considerable attention due to their potential therapeutic and health-promoting benefits. In the present study, a folate-producing probiotic strain, *Lactiplantibacillus plantarum* MGKMVIT11, isolated from fermented fresh peel of *Sechium edule* (chayote), was evaluated for its probiotic characteristics, folate production, and biological activities.

**Methods:**

The probiotic potential of the isolate was assessed through acid and bile tolerance, auto-aggregation, co-aggregation, cell surface hydrophobicity, antibiotic susceptibility, and antibacterial activity analyses. Folate production was optimized using a sequential statistical approach involving one-factor-at-a-time (OFAT) experiments followed by Plackett-Burman (PB) and response surface methodology (RSM) based on central composite design (CCD). Furthermore, the antioxidant and anti-inflammatory activities of the produced folate were evaluated through DPPH radical scavenging assay, reducing power assay, and cytokine expression analysis in LPS-induced U937 cells. The interaction effect of folate in combination with Paclitaxel was also investigated using HCT-116 cells.

**Results and discussion:**

*Lactiplantibacillus plantarum* MGKMVIT11 exhibited significant probiotic properties with high acid tolerance (75.32 ± 0.95%) and bile salt tolerance (78.16 ± 2.53%). The strain demonstrated notable auto-aggregation (70.32 ± 0.95%), co-aggregation (68.51 ± 2.88%), and cell surface hydrophobicity (48.37 ± 4.0%). It also showed susceptibility toward seven antibiotics and strong antibacterial activity against foodborne pathogens, particularly *Salmonella enterica* with a zone of inhibition of 17.1 ± 0.22 mm. OFAT optimization established maltose, peptone, pH 7, 25°C, and 4% inoculum as suitable baseline conditions, while CCD-based RSM identified maltose, ((NH_4_)_2_SO_4_), and MgSO4 as significant variables influencing folate production. Increasing maltose concentration significantly enhanced folate yield. Under optimized conditions, folate production reached 364.35 μg/mL at 24 h, representing 98% agreement with the predicted value (370.99 μg/mL) and a 1.64-fold enhancement compared with unoptimized conditions. In addition, the produced folate exhibited considerable antioxidant activity through enhanced DPPH radical scavenging and reducing power. Anti-inflammatory activity was confirmed by decreased IL-6 and TNF-*α*, levels along with a slight increase in IL-10 expression in LPS-treated U937 cells. Furthermore, the combination of folate with Paclitaxel demonstrated promising interaction effects against HCT-116 cells, suggesting its potential therapeutic applicability.

## 1 Introduction

Folate, a water-soluble vitamin (B9), is a vital nutrient for numerous bodily functions, notably DNA synthesis, cell division, and the prevention of birth defects. Since humans cannot synthesize folate, they must acquire it through their diet (Megala and Kavitha, 2025). Globally, folate deficiency remains a serious public health concern, especially in low-income nations and among vulnerable populations, including pregnant women and the elderly (Rogers et al., 2018). *Lactiplantibacillus plantarum* strains are well recognized for their ability to survive gastric transit and establish themselves in the gut, where they confer health benefits such as antioxidant activity, antimicrobial effects, and cholesterol reduction (Echegaray et al., 2023). They also demonstrate strong acid and bile salt tolerance, a key trait for persistence during gastrointestinal passage (Singhal et al., 2021). This resilience enables fermentation with *Lactobacillus plantarum GX* and *Lactobacillus plantarum GZ,* which significantly boost the nutritional quality of foods by increasing folate and iron content while reducing antinutrients such as phytic acid and tannins that impair nutrient absorption (Ju et al., 2023). In this study, *Lactiplantibacillus plantarum, Levilactobacillus brevis*, and *Lacticasebacillus paracasei* were identified as core lactic acid bacteria for chayote fermentation due to their superior flavor development. Importantly, the microbial-derived folate supplement was found to be safe, unlike synthetic folic acid, which can mask vitamin B12 deficiency and contribute to cardiovascular disorders (Tamene et al., 2023). These findings suggest that microbial folate offers a promising strategy for fortifying staple foods such as rice, cereals, and dairy products (Mahara et al., 2023). Microbially derived folates can enhance host folate status and mitigate the biochemical consequences of deficiency by supporting one-carbon metabolism, nucleotide biosynthesis, and methylation pathways. These fundamental processes regulate endothelial function, neurotransmitter production, and epigenetic mechanisms, thereby underscoring the therapeutic potential of folate-producing probiotic strains (Liu et al., 2022). Although genomic and experimental evidence suggest that folate biosynthesis is common among gut commensals, including *Lactiplantibacillus* and *Bifidobacterium* species, clinical studies providing consistent evidence of therapeutic benefit from targeted probiotic folate delivery in humans remain scarce (D’aimmo et al., 2023). Furthermore, fermentation was optimized using RSM to maximize the recovery of beneficial compounds, particularly pyruvic acid and folate (Zhang and Gao, 2007). This statistical approach allowed for precise control of variables including temperature, pH, and fermentation time, thereby enhancing probiotic efficacy. While the study highlights the probiotic potential of *L. plantarum*, it is essential to consider the strain-specific nature of these benefits. Different strains may exhibit varying levels of health-promoting activities, highlighting the importance of thorough characterization and optimization to maximize their potential in functional food applications (Gökmen et al., 2024; Jain et al., 2025).

Antioxidants are essential molecules that neutralize reactive free radicals, thereby halting the oxidative chain reactions that lead to cellular damage. By neutralizing these oxidative processes, antioxidants lower the incidence of chronic pathologies, including malignancies, cardiovascular dysfunction, and neurodegenerative disorders including Parkinson’s disease and Alzheimer’s disease. They operate through various chemical pathways, including hydrogen donation, radical scavenging, and singlet oxygen quenching (Irivibulkovit et al., 2018). In addition, the radical scavenging potential of folic acid was computationally modeled using DFT calculations reported in a previous study. By evaluating the mechanisms involving single electron transfer (SET) alongside hydrogen atom transfer (HAT), folic acid was identified as a potent antioxidant, specifically through hydrogen donation at the C19-H position. The thermodynamic feasibility of scavenging HOO∙ radicals was then established through potential energy surface analysis, confirming the molecule’s efficacy as a radical scavenger (Nga et al., 2018). Inflammation is a vital adaptive response to pathogens and stimuli; however, while acute inflammation maintains homeostasis, chronic persistence drives various clinical pathologies. Central to this process is the activation of monocytes and macrophages, which release pro-inflammatory cytokines in response to triggers like lipopolysaccharide (LPS) (Samblas et al., 2018). While folate is known to regulate endothelial function and DNA stability, its direct influence on the cytokine storm associated with chronic inflammation requires further investigation (Jones et al., 2019). Our research validates the anti-inflammatory hypothesis by showing that folate treatment effectively ameliorates the imbalance of cytokines in LPS-challenged U937 cells. Drug combinations are the mainstay in treating complex diseases like cancer and chronic inflammation, often aiming for synergy, where the combined effect exceeds the sum of individual potencies. By achieving synergy, therapeutic efficacy can be maintained at lower doses, thereby minimizing adverse side effects. To quantify these interactions, observed responses are compared against null reference models such as HSA, Bliss, Loewe, and ZIP. These models distinguish between synergistic (greater than expected), additive, or antagonistic (lower than expected) interactions based on distinct mathematical assumptions of independence and dose equivalency (Fuentealba-Manosalva et al., 2023).

To address this, researchers have explored the potential of probiotic cultures, particularly lactic acid bacteria (LAB), to produce folate during fermentation, offering a natural and sustainable strategy to enhance dietary intake. In the present study, a novel folate-producing *Lactiplantibacillus plantarum* MGKMVIT11 was isolated from the fermented peel of *Sechium edule* (Chayote); this represents a unique isolation source that has not been previously documented in the literature. Folate was extracted using the tri-enzyme extraction method, and its concentration was determined by a microbiological assay employing the auxotrophic mutant strain *Lactobacillus rhamnosus* ATCC 7469. Since folate biosynthesis is significantly influenced by fermentation parameters such as pH, temperature, and inoculum size, it is critical to optimize the culture medium composition. This work represents the earliest attempt to optimize folate production of the strain using Plackett–Burman (PB) and RSM. Furthermore, the strain’s probiotic potential was evaluated, including an investigation into its antioxidant properties via DPPH radical scavenging and reducing power assays. Subsequently, immunomodulatory responses were assessed by analyzing TNF-*α*, IL-6, and IL-10 expression in LPS-induced U937 cell lines. Finally, the study uniquely demonstrates an overall additive interaction profile of folate in combination with Paclitaxel against HCT-116 cell lines.

## 2 Methodology

### 2.1 Materials

The auxotrophic strain *Lactobacillus rhamnosus* ATCC 7469 was procured in a cryopreserved state from the ATCC (Manassas, VA, United States). Microorganism cultivation was performed using de Man, Rogosa, and Sharpe (MRS) media, including both agar and broth serving as the primary growth media, standard folic acid, antibiotic discs, Folic acid casei medium (FACM), bromocresol purple, potassium ferrocyanide, 2,2-diphenyl-1-picrylhydrazyl (DPPH), L-ascorbic acid, ferric chloride, and Mueller–Hinton broth, all of which were procured from Hi-Media Laboratories, Mumbai, India. The remaining reagents in this study were analytical grade. Test pathogens, including *Bacillus cereus* (MTCC 1272), *Salmonella enterica* (MTCC 1164), and *Shigella flexneri* (MTCC 1457), were obtained from MTCC, Institute of Microbial Technology, Chandigarh, India.

### 2.2 Isolation of folate-producing probiotic bacteria from different food sources

Apple, banana, chayote, cucumber, mung bean sprouts, papaya, and sapota were collected from Vellore district, Tamil Nadu, India. The fresh peels of the samples were washed, homogenized, and fermented for 3 to 5 days at room temperature. Fermented samples were aseptically diluted across concentrations (10^−1^ to 10^−6^) using Milli-Q water. Subsequently, 100 μL of each dilution was inoculated onto MRS agar plates employing the spread-plate technique, followed by incubation at room temperature for 24–48 h. Distinct colonies were selected based on morphological traits and further analyzed via biochemical characterization. Identified isolates were maintained at −80 °C in 50% (v/v) glycerol for long-term preservation.

### 2.3 Preliminary screening for folate-producing probiotic bacteria using bromocresol purple as an indicator

To screen for folate biosynthesis, individual colonies from MRS agar were transferred into 2 mL of FACM and conditioned for 18 h at room temperature. Strains capable of proliferating in this folate-deficient medium were identified as potential producers, with biomass accumulation quantified by measuring the optical density at 600 nm (Hasini Panda et al., 2013). Furthermore, to confirm, the plate assay method was performed for the isolates using Bromocresol purple as an indicator, which was incorporated into FACM adjusted to a pH of 6.8 and incubated for 24 h at 37° C. Folate-producing strains were identified by the transition of the culture medium from purple to yellow. This observable change confirms the metabolic activity and successful folate synthesis by the specific strain (Jiang et al., 2024). The selected isolates were then taken for 16S rRNA sequencing.

### 2.4 Folate quantification by microbiological assay

Folate quantification was performed using a modified microbiological assay, with the cryoprotected folate auxotrophic mutant *Lactobacillus rhamnosus* ATCC 7469 serving as the indicator organism. The cultures grown overnight in MRS broth were centrifuged at 5500 rpm for 5 min, washed three times with sterile saline (0.85%), and resuspended in saline solution. A 2% inoculum of the suspension was transferred into freshly prepared double-strength folic acid casei medium (2 x FACM). Sample extracts (100 μL) were dispensed into 96-well microplates, followed by the addition of 100 μL of indicator culture (4% v/v) prepared in 2 x FACM supplemented with chloramphenicol (20 μg/mL) to prevent contamination. Folic acid standards were prepared at concentrations of 20, 40, 60, 80, and 100 μg/mL to construct the calibration curve. Plates were incubated at 37 °C for 18 h, and bacterial growth was measured spectrophotometrically at 420 nm. Folate concentrations in samples were calculated from the standard calibration curve (Laiño et al., 2012).

### 2.5 Characterization of potent strain

The folate-producing isolate was characterized based on its morphological, physiological, and biochemical traits in accordance with Bergey’s Manual of Determinative Bacteriology. For molecular identification, 16S rRNA gene sequencing and phylogenetic analysis were performed, with the gene amplified using universal primers (Forward: 5′- GGATGAGCCCGCGGCCTA-3′ and Reverse: 5′ – CGGTGTGTACAAGGCCCGG-3′) sourced from Biokart India Pvt. Ltd., Bangalore, India. To amplify the target DNA, a 50 μL PCR mixture was prepared using 10 μL of 10 x Taq DNA polymerase (3 U/mL), along with 4 μL of dNTPs (2.5 mM), and 2 μL of each specific primer, along with 1 μL of the DNA template. The thermal profile began with denaturation (94 °C, 3 min), followed by 30 cycles consisting of denaturation (94 °C, 1 min), annealing (50 °C, 1 min), extension (72 °C, 2 min), and a final extension (72 °C, 7 min). After confirming the ~1,500 bp products via 1% (m/v) agarose gel electrophoresis at 150 V, sequencing was executed on an ABI 3130 Genetic Analyzer using Big Dye Terminator version 3.1. Finally, 16S rRNA sequences were compared against the GenBank database using BLAST, with a Jukes–Cantor model-based weighted neighbor-joining tree used for phylogenetic analysis.

### 2.6 Extraction of folate from the strain

of cultivation in MRS broth, the biomass was harvested via centrifugation at 5500 g for 10 min. The resulting clear liquid was decanted, and the cell pellet underwent three wash cycles with 0.85% NaCl solution. The pellet was then resuspended in NaCl and mixed well. From the pellet sample, 1 mL was taken and mixed well with an equal volume of protective buffer. Protective buffer preparation: 0.1 M potassium phosphate buffer with a pH of 6.8, enriched with 1% (m/v) ascorbic acid to inhibit vitamin oxidation. The supernatant was reserved after centrifugation (10,000 g, 10 min) as the extracellular folate fraction. To isolate intracellular folate, the remaining cell pellet was resuspended in 1 mL of the protective buffer. Both fractions were heated for 5 min at 100 °C, rapidly cooled, and then centrifuged (14,000 g, 10 min). The cell debris was removed, and the supernatants were collected (Laiño et al., 2019). The 1 mL of intracellular sample was treated with protease derived from *Streptomyces griseus* at a concentration of 2 mg/mL (Sigma, St. Louis, MO, USA). After thorough blending, the mixture was stored in the dark for 2 h at 37 °C. After 2 h, the enzyme was heated to inactivate it at 100 °C for 5 min in a water bath and then cooled immediately. The sample was subsequently mixed with 20 mg/mL of *α*–amylase from *Aspergillus oryzae* (Sigma Chemical, St. Louis, MO, USA), and 100 μL of chicken pancreas was added. Following a 2-h incubation in the dark, the sample was centrifuged (5,000 g, 10 min) (Sallam et al., 2021). The upper phase was collected and preserved at −80° C, while the pellet was discarded.

### 2.7 UPLC analysis

The standard folate (5 μg/mL) was dissolved in sterile Milli-Q H_2_O. Since folate is a water-soluble compound, it was completely dissolved and filtered using a sterile 0.22 μm syringe filter for the sample and standard. For chromatographic analysis, 2 μL of the sample was injected into a UPLC (Acquity UPLC H-class) system with a PDA eλ detector. The separation was carried out on a C18 column (4.6 mm x 250 mm x 5 μm). A gradient mobile phase was composed of (A) 0.1% (v/v) aqueous formic acid and (B) methanol. The gradient profile began with a 1-min hold at 90% A, followed by a linear reduction to 50% A over 2 min. The concentration was then returned to 90% A over 1 min and maintained for 2 min to allow for column re-equilibration (El-Hawiet et al., 2022). A total run time of 15 min was employed, with the column temperature maintained at 40 °C, and the absorbance was monitored at 283 nm.

## 3 Probiotic properties of folate-producing strain

### 3.1 Analysis of hemolytic potential

To evaluate the hemolytic capacity of the isolates, the agar plates were supplemented with 5% sheep blood, after which fresh cultures were streaked and incubated for 24–48 h at 37 °C. The plates were then examined for the presence or absence of zone clearing. *Staphylococcus aureus* ATCC 25923 served as the positive control due to its characteristic *β*-hemolytic activity (Tarrah et al., 2018).

### 3.2 Acid and bile tolerance

For the acid tolerance assay, cells from a culture grown overnight in MRS broth were collected via centrifugation. The resulting pellet was washed twice with PBS buffer (pH 7.0) and subsequently resuspended. Experimental trials were conducted by adding 0.5 mL of the prepared suspension to 4.5 mL of MRS broth adjusted to pH 2.5, with MRS broth at pH 6.5 serving as the reference control. Viable bacteria were enumerated at the 2nd, 3rd, and 4th h by plating on MRS agar and incubating at room temperature for 24 h (Divyashree et al., 2021).

Bile tolerance was assessed by inoculating 0.5 mL of the bacterial suspension into 4.5 mL of MRS broth containing 0.3% bile salt. A reference control was maintained in bile-free MRS medium. At intervals of 2, 3, and 4 h during incubation, aliquots were removed, diluted serially, and spread onto MRS agar plates. These plates were then incubated at room temperature for 24 h to determine the concentration of surviving cells. Viable cell counts were expressed as logarithmic values of colony-forming units per milliliter (log CFU/mL) (Pieniz et al., 2014; Goyal et al., 2026). The percentage of acid and bile tolerance was calculated using a specific formula.


$$\text{Survival rate}\;(\%)=\frac{\text{Final}\;(\;\mathrm{CFU}/\mathrm{mL})}{\text{Initial}\;(\;\mathrm{CFU}/\mathrm{mL})}\times 100$$

### 3.3 Auto-aggregation and co-aggregation

Overnight culture grown in MRS broth was collected by centrifugation (8,000 rpm, 10 min) at 4 °C. The cell pellets were washed twice with PBS (pH 7.4) and resuspended in the same buffer to an initial OD (A₀) of 0.6 at 600 nm. Following incubation at room temperature, the auto-aggregation was assessed by observing the absorbance of the upper layer at 3, 6, and 24 h (Pessoa et al., 2017). The percentage of auto-aggregation was determined by the following equation:


$$\text{Autoaggregation}\;(\%)=\frac{A0-\mathrm{At}}{A0}\times 100$$

where A0 is the initial absorbance at 0th h and At is the final absorbance of 3, 6, and 24 h incubation.

According to Li et al. (2020), the co-aggregation ability of the selected isolate was evaluated by combining equal volumes with pathogenic strains (*Bacillus cereus* MTCC 1272, *Salmonella enterica* MTCC 1164, *Shigella flexneri* MTCC 1457, and *Staphylococcus aureus* 25923). After being resuspended in PBS as described for the auto-aggregation assay, the initial optical density (A₀) of these mixed cultures was standardized to 0.6 at 600 nm. Following 24 h of cultivation at room temperature, the extent of co-aggregation was measured by monitoring the absorbance of the upper suspension at 600 nm at 3, 6, and 24 h. The percentage of co-aggregation was then calculated based on these measurements.


$$\mathrm{Co}-\text{aggregation}(\%)=\frac{(\text{Apro}+\text{Apat})-\text{Amix}}{\;\text{Apro}+\text{Apat}}\times 100$$

Here, Apro and Apat denote the individual absorbances of the isolate and pathogenic strain suspensions, respectively, while Amix represents the absorbance of their combined suspension.

### 3.4 Surface hydrophobicity assay

Cell surface hydrophobicity was determined by harvesting MRS broth cultures via centrifugation. The cells were then washed twice and resuspended in PBS to a standardized initial A_600_ of 0.6. Following exposure of cell suspensions to chloroform, ethyl acetate, and xylene, the mixtures were vortexed, held at 37 °C for 1 h, and the absorbance of the aqueous phase was recorded at 600 nm (Mladenović et al., 2020). This value was then used in a specific formula to determine the cell surface hydrophobicity.


$$\text{Cell surface hydrophobicity}\;(\%)=1-\frac{A1}{A0}\times 100$$

The variable A₀ signifies the absorbance recorded at the initial time (0 h), and A₁ signifies the absorbance recorded after a duration of 1 h.

### 3.5 Antibiotic sensitivity assay

Antibiotic susceptibility was evaluated using the Kirby–Bauer disc diffusion assay. The isolate was cultured in MRS broth and calibrated to a 0.5 McFarland turbidity standard. The isolate was evenly distributed across MRS agar plates using the lawn technique, followed by placing standard antibiotic discs onto the surface: chloramphenicol (C; 30 mcg), erythromycin (E; 15 mcg), gentamicin (GEN; 10 mcg), rifampicin (RIF; 5 mcg), tetracycline (TE; 30 mcg), ampicillin (AMP; 10 mcg), and streptomycin (S; 10 mcg). Following 24 h of incubation, the clear zones around the antibiotic discs were measured (mm) to evaluate the isolate’s susceptibility. The susceptibility was categorized as resistant (<8 mm), intermediate (8–10 mm), and susceptible (>10 mm) according to established criteria (Mehmood et al., 2024).

### 3.6 Antibacterial efficacy

Agar well diffusion was employed to evaluate the antibacterial activity of the isolate. The isolate was cultured overnight in MRS broth, while the test pathogens—*Bacillus cereus*, *Salmonella enterica*, and *Shigella flexneri*—were grown overnight in Mueller–Hinton broth, with all cultures incubated at 37 °C. Following incubation, the isolate was harvested, and cell-free supernatants were sterilized by passage through a 0.22-μm membrane. Mueller–Hinton agar plates were inoculated with the pathogens using the lawn culture technique, and the agar was perforated with 5 mm wells using a sterile cork borer, which were filled with 100 μL of the sterile supernatant. After 24 h of incubation, the inhibition zones were observed and graded as weak (7–9 mm), intermediate (10–13 mm), or strong (14–24 mm) (Mulaw et al., 2019; Arora et al., 2024).

## 4 Optimization of process parameters for enhanced production of folate

### 4.1 OFAT analysis

Preliminary screening of medium components and culture conditions was carried out using OFAT analysis. To enhance folate production, five major factors were analyzed in this study: carbon sources, nitrogen sources, temperature, pH, and inoculum size. To evaluate alternative carbon utilization, glucose was replaced in the basal medium with fructose, galactose, maltose, sucrose, and lactose. Likewise, the standard nitrogen source, protease peptone, was substituted with yeast extract, casein, tryptone, peptone, and soy peptone. The temperature was varied between 25 and 45 °C for optimization. The optimal pH for folate production was explored across a range of 3 to 8. Inoculum sizes ranging from 2 to 10% were also tested to enhance folate production (Hemalatha and Subathra Devi, 2022). After the incubation periods, the folate was extracted, and concentrations were determined using a microbiological assay.

### 4.2 Significant variable identification via Plackett–Burman design

Plackett–Burman (PB) design was employed to identify the impact of key medium components on the folate production capability of the target strain. Eleven variables were investigated at two-level factorials, including low (−1) and high (+1) across twelve experimental conditions generated by JMP Pro 18 Software. The trials were conducted in 30 mL of medium (pH 7) and incubated at 25 °C for 24 h and 48 h, respectively. Following this, a microbiological assay was performed to evaluate folate concentration, while the experimental data were analyzed using a first-order polynomial model:


$$Y=\beta_{0}+\Sigma \beta_{i}X_{i}$$

where *Y* represents the predicted response (folate concentration), *β_0_* is the intercept, *β_i_* represents the regression coefficients, and *X_i_* denotes the independent variables. The model was used to evaluate the individual effects of each variable on folate production. All subsequent experiments were carried out in triplicate, and the mean folate concentration was considered the response value. Multiple linear regression analysis was performed to assess the relationship between independent variables and the response (Abo-Zaid et al., 2023).

### 4.3 Statistical optimization of folate using response surface methodology

RSM was utilized to enhance folate production by determining the optimal concentrations and interactive effects of key variables. Based on the PB design results, ammonium sulfate, maltose, and magnesium sulfate were selected for further study using a central composite design (CCD) generated by JMP Pro 18 software (Luong et al., 2023). The CCD consisted of 20 experimental runs, including six center point replicates. The selected variables were evaluated at three distinct levels: low (−1), medium (0), and high (+1), determined based on OFAT screening results. The actual levels used were ammonium sulfate (A) at 10, 15, and 20 g/L; maltose (B) at 10, 20, and 30 g/L; and magnesium sulfate (C) at 0.5, 0.75, and 1.0 g/L.

Based on the findings of the OFAT analysis, temperature, pH, and inoculum size were maintained at constant levels throughout the subsequent experiments. Conversely, glucose, ferrous sulfate, and NaCl were excluded from the culture medium due to their negative regression coefficient values observed in the earlier analysis. All procedures were conducted in 100 mL flasks filled with 30 mL of medium at pH 7, as per the design protocol. Following incubation for 48 h at 25 °C, the folate concentration was recorded as the response.


$$\begin{array}{l} Y=\beta_{0}+\beta ₁X₁+\beta ₂X₂+\beta ₃X₃+\beta ₁₂X₁X₂+\beta ₁₃X₁X₃+\beta ₂₃X₂X₃\\ +\beta ₁₁X{₁}^{2}+\beta ₂₂X{₂}^{2}+\beta ₃₃X{₃}^{2} \end{array}$$

The dependent variable, folate production (Y), was modeled as a function of three independent variables: ammonium sulfate (X1), maltose (X2), and magnesium sulfate (X3). The model incorporated an intercept (*β_0_*), linear (*β_1_-β_3_*), interaction (*β_12_, β_13_, β_13_*), and quadratic (*β_11_, β_22_, β_33_*) coefficients. Analysis of variance (ANOVA) was performed on the response data using JMP Pro 18 Software to assess the overall model significance (*F*-value and *p*-value), lack of fit, and goodness of fit (*R^2^* value). The fitted equation was subsequently applied to construct a 3D response surface plot, enabling the identification of optimal concentrations and interaction effects among the independent variables (Johal et al., 2025).

## 5 Bioactivity assays of folate

### 5.1 Antioxidant activity

The folate antioxidant potential was assessed through DPPH radical scavenging. For the assay, varying folate concentrations were prepared, ranging from 2 to 10 μg/mL. From each concentration, 1 mL of an aliquot was mixed with DPPH solution (0.5 mM) and Milli-Q H2O. This reaction mixture was maintained at room temperature for 30 min in the dark, after which absorbance was measured at 517 nm. As a positive control, ascorbic acid (2–10 μg/mL) was used. The percentage of free radical scavenging was formulated by the equation:


$$\text{DPPH activity}\;(\%)=1-\frac{\left(\text{Asample}\right)}{\left(\text{Ablank}\right)}\times 100$$

wherein the blank comprised Milli-Q H_2_O and DPPH solution, while A represented the absorbance of the reaction mixture containing Milli-Q water and the sample.

The reductive capacity of folate was quantified by measuring the transformation of Fe^3+^ to Fe^2+^. Reaction mixtures containing 1.5 mL of folate (2–10 μg/mL), phosphate buffer, and potassium ferrocyanide were incubated (50 °C, 25 min) and subsequently acidified with 10% TCA. After centrifugation (3,000 x g, 10 min), the upper layer was reacted with 0.1% ferric chloride. The resulting Prussian blue complex was obtained spectrophotometrically at 700 nm. As a positive control, ascorbic acid was used (Angelin and Kavitha, 2023; Khanna et al., 2025).

### 5.2 Anti-inflammatory activity

#### 5.2.1 Evaluation of cytotoxicity

The human monocyte-derived U937 cell line was obtained from NCCS, Pune, and cultured in RPMI 1640 medium supplemented with 10% FBS. Cells were maintained at 37 °C in a humidified atmosphere containing 5% CO_2_ with regular passaging to maintain a confluence of 80–90%. The effect of folate on U937 cell viability was assessed using the MTT assay. Cells were plated at a density of 10×10^3^ per well in a 96-well plate for 48 h. After treatment, 10 μL of MTT reagent (5 mg/mL) was dispensed into each well and maintained for 4 h at 37 °C to allow for formazan crystal formation. These crystals were then dissolved in 100 μL of isopropanol containing 1 N HCl by gently agitating for 20 min, and absorbance was recorded at 570 nm using an Agilent BioTek Cytation 5 microplate reader (Olsen et al., 2014). The percentage of cell viability was calculated using the formula:


$$\text{Cell Viability}\;(\%)=\frac{\mathrm{OD}\;(\text{Treated})}{\mathrm{OD}\;(\text{Control})}\times 100$$

#### 5.2.2 Rt-PCR

U937 cells were maintained in 6-well plates (1 × 10^6^ cells/well) using RPMI 1640 medium containing 1% FBS. To evaluate immunomodulatory activity, inflammation was induced using lipopolysaccharide (LPS; 100 ng/mL) for 24 h. The anti-inflammatory effect of folate (100 μg/mL) was assessed by comparing cytokine gene expression between the control group (LPS-stimulated cells) and the treated group (cells co-treated with LPS and folate). The resulting expression levels of TNF-*α*, IL-6, and IL-10 were analyzed to determine the modulatory effect of folate under inflammatory conditions (Sukjamnong and Santiyanont, 2015).

#### 5.2.3 RNA extraction and cDNA synthesis

Total RNA was derived from both the treated and control cells after 24 h of treatment using TRIzol reagent according to the manufacturer guidelines. The obtained RNA was then quantified and checked for purity using a NanoDrop 1000 spectrophotometer. Subsequently, the RNA was converted into complementary DNA (cDNA) through reverse transcription using the G-Bioscience synthesis kit.

#### 5.2.4 Quantitative real-time PCR (qRT-PCR)

qRT-PCR was conducted to quantify mRNA expression using gene-specific primers for TNF-α (Forward: 5’-CCTCTCTCTAATCAGCCCTCTG-3′, Reverse: 5-GAGGACCTGGGAGTAGATGAG-3′), IL-6 (Forward: 5’-ACTCACCTCTTCAGAACGAATTG-3′, Reverse: 5-CCATCTTTGGAAGGTTCAGGTTG-3′), IL-10 (Forward: 5’-ACGGCGCTGTCATCGATT-3′, Reverse: 5-ATTGCATCTGGCAACCCTAC-3′), and *β*-actin (Forward: 5′- ATGATATCGCCGCGCTCG-3′, Reverse: 5 CGCTCGGTGAGGATCTTCA-3′), which served as a housekeeping gene for normalization. Amplification conditions included an initial denaturation (95 °C, 2 min), followed by 40 cycles of denaturation (95 °C, 10 s), annealing (60 °C, 20 s), and extension (72 °C, 15 s), concluding with a final extension at 72 °C for 5 min (Biotek, Agilent). Relative mRNA abundance was determined using the comparative ΔΔC_t_ method, and expression levels were standardized to β-actin, and quantified as fold variations relative to the LPS-stimulated control group (Menk et al., 2015).

### 5.3 Anti-cancer activity

#### 5.3.1 Growth conditions of HCT-116

Human colorectal carcinoma (HCT-116) cells were purchased from NCCS, Pune, and maintained in DMEM supplemented with 25 mM glucose and 10% (v/v) FBS in T-25 flasks, grown at 37 °C in a humidified 5% CO_2_ environment until reaching 80% confluency. Upon reaching confluency, cells were harvested by trypsinization, collected by centrifugation, and resuspended in the same culture medium. Subsequently, cells were distributed onto 96-well plates (5 × 10^3^ cell) and maintained at 37 °C under 5% CO_2_ for a 48 h incubation period (Xiang et al., 2020).

#### 5.3.2 Treatment

A stock solution of Paclitaxel (1 μM) and folate (10,000 nM) was prepared in Milli Q water. Both drug solutions were then filtered and serially diluted in DMEM medium supplemented with 25 mM glucose and 1% (v/v) FBS to obtain final concentrations of 1,000, 500, 250, 125, and 62.5 nM for folate and 0.0625, 0.12, 0.25, 0.5, and 1 nM for paclitaxel. Following 48 h of incubation of HCT-116 cells in 96-well plates and removal of cell-free spent medium, 100 μL/well of individual drug solutions or 50 μL + 50 μL/well of the combined drug solutions were added according to the designated plate layout. The cultures were then incubated for a further 24 h at 37 °C in a humidified incubator with 5% CO_2_.

#### 5.3.3 Determination of cell viability in HCT-116

After 48 h of treatment, MTT solution (5 mg/mL) was added to each well containing HCT-116 cells and incubated for 4 h at 37 °C in the dark to allow for the formation of formazan crystals. The medium containing the MTT reagent was then carefully removed, and 100 μL of isopropanol containing 4 mM HCl was added to each well to dissolve the formazan crystals. The plates were gently shaken for 20 min to ensure complete dissolution, and absorbance was measured at 570 nm using an Agilent BioTek Cytation 5 Cell Imaging Multimode Reader. Finally, cell viability (%) was assessed using the formula:


$$\text{Cell Viability}\;(\%)=\frac{\mathrm{ABS}\;(\text{Treated})}{\mathrm{ABS}\;(\text{Control})}\times 100$$

### 5.4 Data analysis

Preliminary data organization was conducted using Microsoft Excel. Drug combination synergy was subsequently evaluated with the SynergyFinder+ platform,^1^ employing the following parameters: data format set to table, response specified as % viability, data annotation enabled, synergy mapping applied, baseline correction set to none, plot type designated as Heat map, and global synergy calculated as the Mean.

### 5.5 Statistical analysis

ANOVA was employed to compare the experimental and predicted response values. The statistical JMP Pro 18 software (SAS Institute Inc., Cary, NC, United States) was utilized for the design evaluation and interpretation of the experimental results. Figures of single-factor experiments were plotted using Origin Pro 2025 software (Northampton, Massachusetts, United States). The threshold for statistical significance was set at *p* < 0.0001.

## 6 Results

### 6.1 Isolation of folate-producing strain

**Figure 1:**
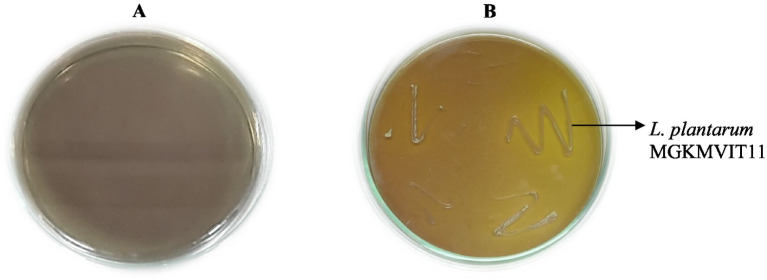
Folate production by LAB on BCP agar (A) Control (B) Test.

**Figure 2:**
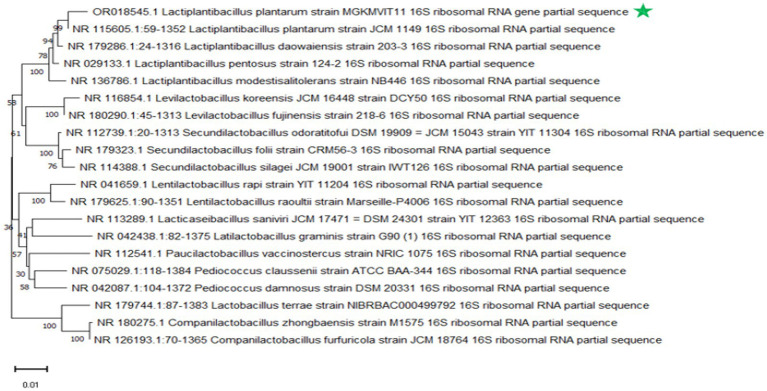
Phylogenetic tree of *Lactiplantibacillus plantarum* MGKMVIT11.

The isolate obtained from the fermented peel of *Sechium edule* (chayote) was recognized as *Lactiplantibacillus plantarum* MGKMVIT11 based on a combination of morphological observations, biochemical profiling, and 16S rRNA gene sequencing. This strain forms gram-positive, rod-shaped colonies, is immobile and non-sporulating, is both oxidase- and catalase-negative, can grow at room temperature, and functions as a facultative anaerobe. The biochemical tests, including indole, methyl red, Voges–Proskauer, and citrate utilization, were all negative; the nitrate test was positive, and the triple sugar iron test exhibited an acid slant/acid butt. Colonies were characterized as medium-sized, round-ended, slightly raised, with a creamy or white texture. Figures 1A,B illustrate the folate-producing potential of the strains on FACM-BCP medium. The transition of the agar color from purple to yellow provides clear evidence of the folate production capabilities. Figure 2 illustrates the phylogenetic analysis of *L. plantarum* MGKMVIT11 based on 16S rRNA gene sequences. This genetic information is available in the GenBank repository via accession number OR018545. The strain has shown 98.62% similarity with *L. plantarum* strain JCM1149.

### 6.2 Extraction of folate and determination

Folate was extracted from the overnight-grown culture in MRS broth at room temperature. Using the tri-enzyme method, the sample was filtered through a sterile 0.22 μm syringe filter, and a microbiological assay was conducted to determine the concentration of folate from a known standard curve of folic acid (Sallam et al., 2021). The folate concentration was obtained to be 222.7647 μg/mL.

### 6.3 UPLC

**Figure 3:**
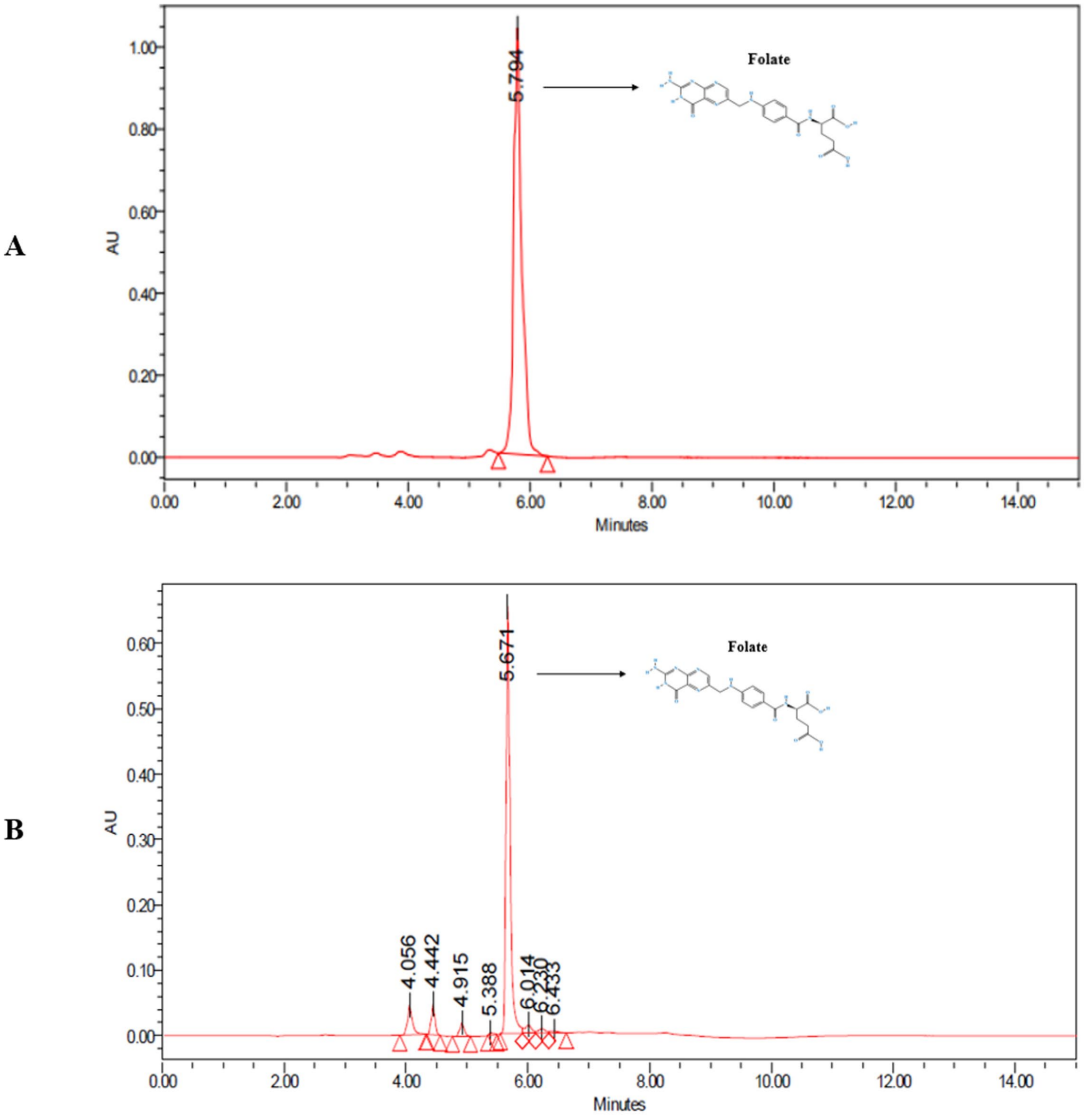
UPLC chromatogram **(A)** standard—folate and **(B)** sample—*L. plantarum* MGKMVIT11.

Folate analysis was performed using UPLC, and Figure 3 shows the chromatograms of both the reference standard and the bacterial folate-containing extract. A chromatographic peak observed at RT 5.671 min was consistent with the retention time of the folate standard (RT 5.794 min), suggesting the presence of folate in the extracted sample. Using an external standard method, the sample peak height (654,112) was used to estimate a concentration of 1.59 μg/mL relative to a 5 μg/mL folate standard peak (1,040,174).

### 6.4 Hemolytic assay

**Figure 4:**
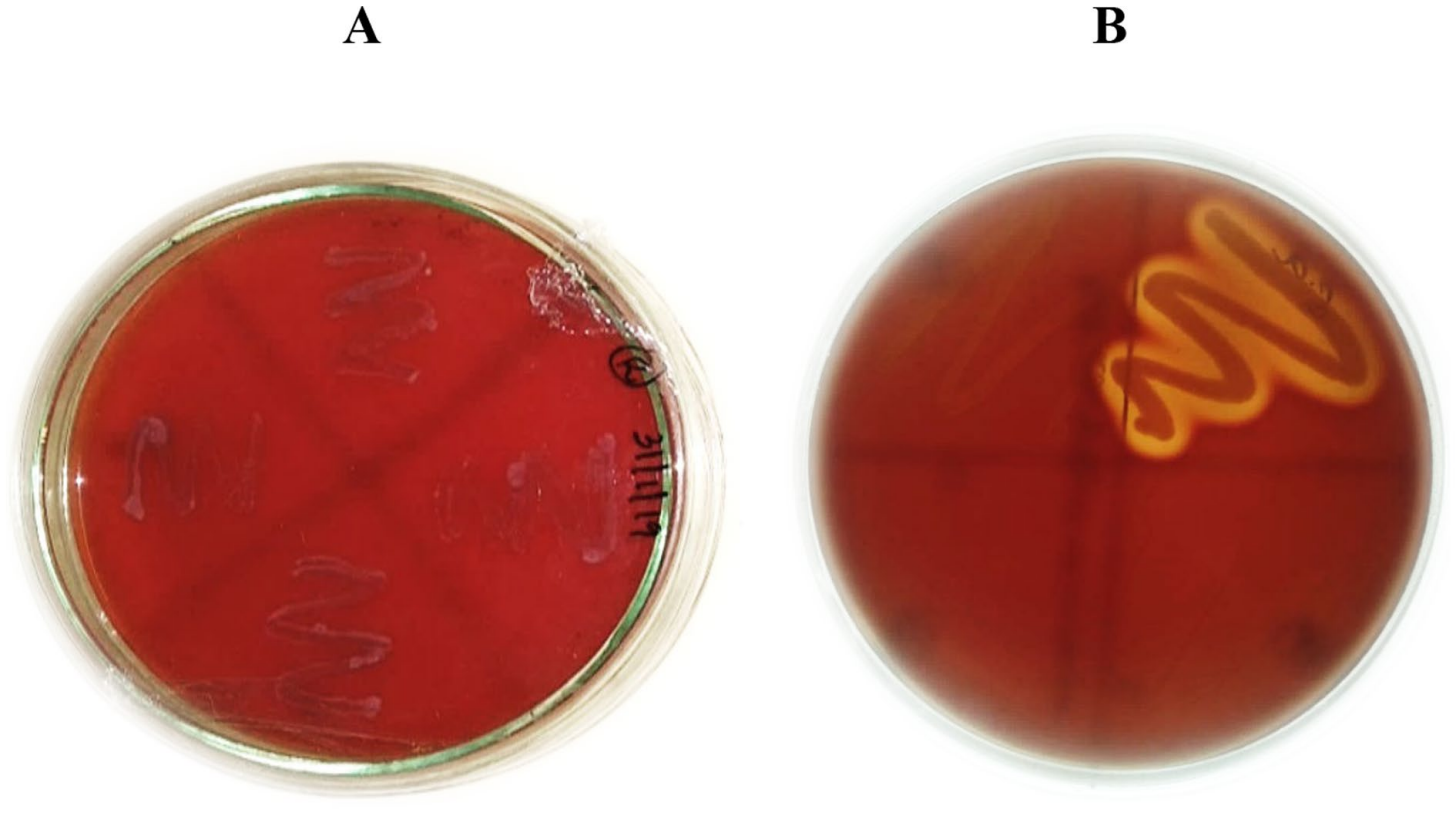
Hemolytic assay **(A)** Test—*L. plantarum* MGKMVIT11 **(B)** Control—*Staphylococcus aureus* 25923.

*L. plantarum* MGKMVIT11 is *γ*-hemolytic (non-hemolytic), indicating it does not lyse red blood cells in the assay. Conversely, *Staphylococcus aureus* 25923, the positive control, demonstrates *β*-hemolysis, characterized by complete red blood lysis, confirming the validity of the hemolysis assay. This non-hemolytic characteristic of the strain is an important factor in evaluating its non-toxicity and safety for potential applications (Diez-Gutiérrez et al., 2022). Figure 4 illustrates the hemolytic assay plate.

### 6.5 Acid and bile tolerance

To assess the strain’s ability to survive under the stressful conditions of the gastrointestinal environment, the acid and bile salt tolerance of *Lactiplantibacillus plantarum* MGKMVIT11 was evaluated. The survival rate of the strain was monitored over 4 h of exposure to simulated gastric fluid at low pH (2.5) and synthetic simulated intestinal fluid enriched with 0.3% (w/v) bile acids. Following the 4 h incubation period, the survival percentage of the strain was determined to be 75.32 ± 0.95% under acidic conditions and 78.16 ± 2.53% in the presence of bile salts. These results indicate a substantial capacity of the strain to withstand both low pH and the presence of bile salts, suggesting its potential to survive passage through the upper gastrointestinal tract.

### 6.6 Auto-aggregation, co-aggregation, and cell hydrophobicity

The maximum auto-aggregation ability of the strain is about 70.32 ± 0.95%, with cells adhering to each other to form clumps or aggregates. The low standard deviation suggests that this ability is consistent across replicates. Table 1 presents the percentage of auto-aggregation, co-aggregation, and cell surface hydrophobicity of *Lactiplantibacillus plantarum* MGKMVIT11 (means ± SD). This indicates the cell adhesion potential that leads to biofilm formation. The strain can co-aggregate with *Bacillus cereus*, *Salmonella enterica*, *Staphylococcus aureus*, and *Shigella flexneri* at 61.54 ± 2.0%, 52.24 ± 4.07%, 68.51 ± 2.88%, and 40.41 ± 2.19%, respectively. These results demonstrate that the strain can interact and adhere to several pathogenic bacteria. The varying percentages indicate different levels of affinity for each pathogen, with the highest co-aggregation observed with *S. aureus* and *B. cereus*, and the lowest with *S. flexneri.* The standard deviations provide a measure of the consistency of these interactions. This co-aggregation ability suggests that this specific strain could have potential applications in controlling or inhibiting the growth and colonization of these specific foodborne or enteric pathogens. The cell surface hydrophobicity assay, using different solvents, provides insights into the nature of the bacterial cell surface. A higher affinity, indicated by a higher percentage with a particular solvent, suggests a greater similarity in surface properties. The hydrophobicity analysis demonstrated that this strain exhibits an enhanced affinity for xylene (non-polar solvent) at 48.37 ± 4.0%, indicating that the cell surface exhibits a significant degree of hydrophobicity (water-repelling) compared to chloroform (acidic solvent) at 32.14 ± 3.0%, which has a lower affinity, suggesting a less pronounced presence of basic or positively charged components on the cell surface, and ethyl acetate (basic solvent) at 38.0 ± 2.1%, which has an intermediate affinity, indicating the presence of some acidic or negatively charged components, but less dominant than the hydrophobic characteristics.

**Table 1:** Percentage of auto-aggregation, co-aggregation, and cell surface hydrophobicity of *Lactiplantibacillus plantarum* MGKMVIT11.

Strain	Auto –aggregation (%)	Co-aggregation (%)				Cell surface hydrophobicity (%)		
		*B. cereus*	*S. enterica*	*S. aureus*	*S. flexneri*	Chloroform	Ethyl acetate	Xylene
*Lactiplantibacillus plantarum* MGKMVIT11	70.32 ± 0.95	61.54 ± 2.0	52.24 ± 4.07	68.51 ± 2.88	40.41 ± 2.19	32.14 ± 3.0	38.0 ± 2.1	48.37 ± 4.0

### 6.7 Antibiotic susceptibility test

**Figure 5:**
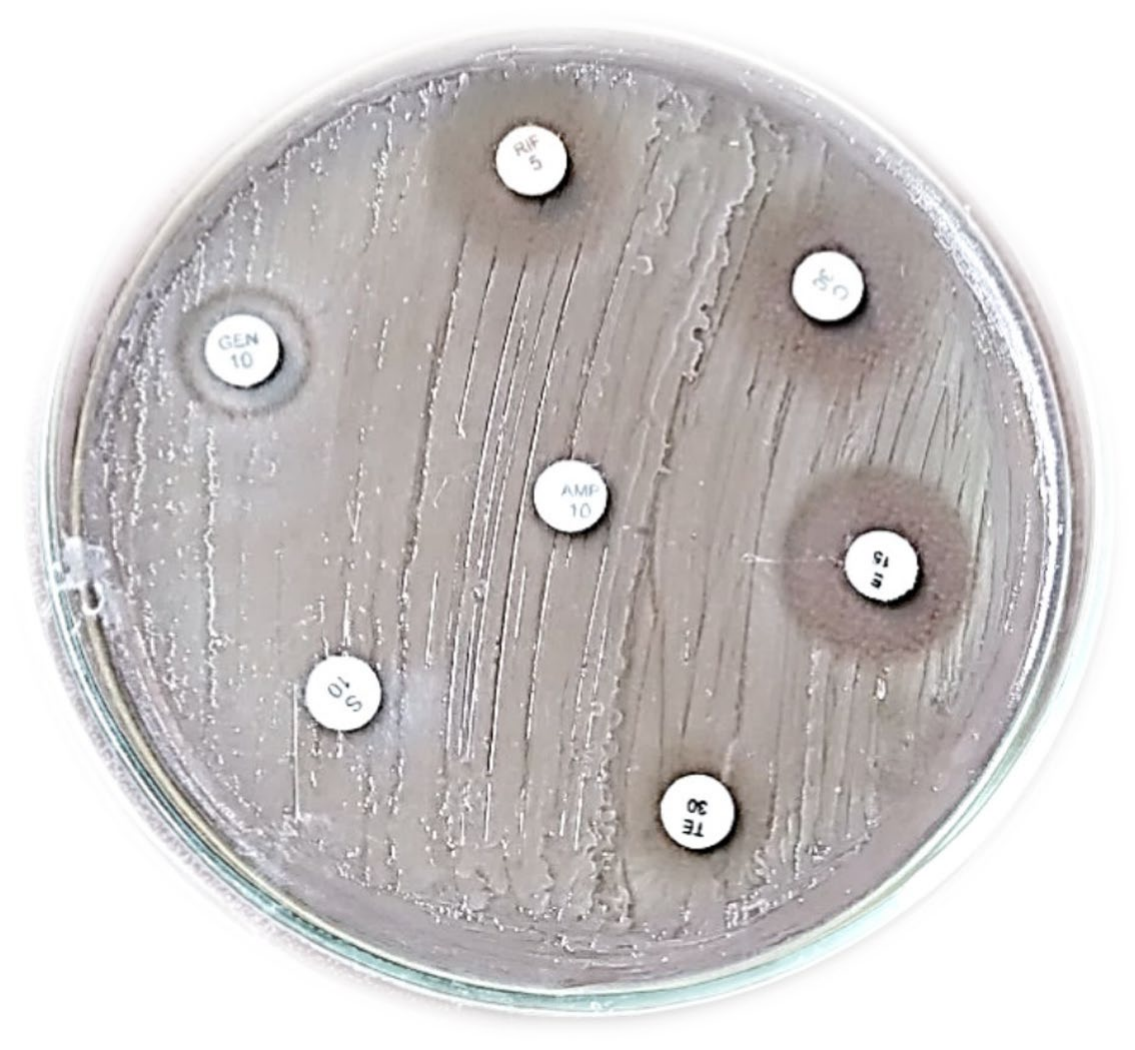
Antibiotic susceptibility of *Lactiplantibacillus plantarum* MGKMVIT11.

Despite the Generally Recognized as Safe (GRAS) classification of LAB, a thorough evaluation of their intrinsic capacity to interact with diverse antibiotics is crucial. This proactive evaluation is essential to mitigate the risk of these bacteria acting as reservoirs or vectors for the transmission of antibiotic resistance genes within microbial communities. Table 2 shows that antibiotic susceptibility testing revealed that *Lactiplantibacillus plantarum* MGKMVIT11 exhibited a moderate zone of inhibition to chloramphenicol, erythromycin, and rifampicin, suggesting that the strain is likely susceptible to these antibiotics. However, tetracycline showed a very small zone of inhibition, indicating minimal to no effect on the growth of this strain. Conversely, the isolate demonstrated complete resistance to ampicillin and streptomycin, suggesting the presence of inherent or acquired resistance mechanisms against these antibiotics. Furthermore, the zone of inhibition observed for gentamicin was interpreted as susceptible; the comparatively smaller inhibition zone relative to chloramphenicol and erythromycin suggests lower sensitivity, which may warrant further investigation into potential sub-inhibitory effects or the development of resistance under prolonged exposure. Figure 5 illustrates the antibiotic susceptibility of *Lactiplantibacillus plantarum* MGKMVIT11 against various antibiotic discs.

**Table 2:** Antibiotic susceptibility of *Lactiplantibacillus plantarum* MGKMVIT11.

S. no	Antibiotic disc	Concentration (μg/disc)	Zone of inhibition (mm)
1)	Chloramphenicol (C)	30	18.2 ± 0.27
2)	Erythromycin (E)	15	17 ± 0.42
3)	Gentamicin (GEN)	10	12.5 ± 0.22
4)	Rifampicin (RIF)	5	18 ± 0.45
5)	Tetracycline (TE)	30	5 ± 0.21
6)	Ampicillin (AMP)	10	0.00 ± 0.05
7)	Streptomycin (S)	10	0.00 ± 0.02

<8 mm resistant, 8–10 mm moderate, and >10 mm susceptible.

### 6.8 Antibacterial activity

**Figure 6:**
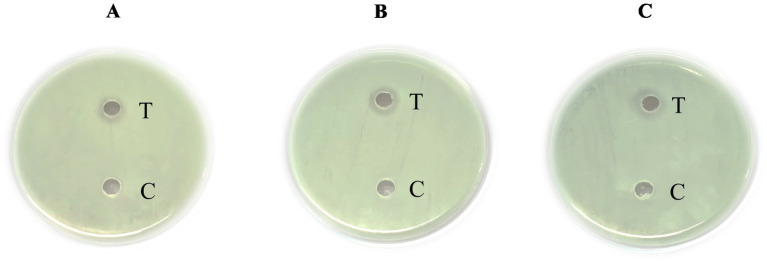
Antibacterial activity of *L. plantarum* MGKMVIT11 **(A)** *Bacillus cereus* **(B)** *Salmonella enterica* **(C)** *Shigella flexneri*. Here, T indicates cell-free supernatant sample and C indicates control, which is sterile Milli Q H_2_O.

The capacity of probiotic-derived bioactive compounds or postbiotics, including organic acids, bacteriocins, and potentially cell surface components, to modulate the host intestinal microbiota is well-documented. Table 3 depicts the antibacterial activity of *Lactiplantibacillus plantarum* MGKMVIT11 against the enteric pathogens *Bacillus cereus*, *Salmonella enterica*, and *Shigella flexneri*. The cell-free supernatant from the strain demonstrated antagonistic activity against all tested enteric pathogens. Notably, the highest inhibitory effect was observed against *Salmonella enterica*, with a zone of inhibition measuring 17.1 ± 0.22 mm. These results suggest the potential of the strain to exert inhibitory effects against common gut pathogens. Figure 6 shows the antibacterial activity of *L. plantarum* MGKMVIT11 against various pathogens.

**Table 3:** Antibacterial activity of *L. plantarum* MGKMVIT11.

S. no	Pathogens	Zone of inhibition (mm)
1)	*Bacillus cereus*	13.4 ± 0.61
2)	*Salmonella enterica*	17.1 ± 0.22
3)	*Shigella flexneri*	10.5 ± 0.53

### 6.9 Optimization of folate production OFAT analysis

#### 6.9.1 Carbon sources

**Figure 7:**
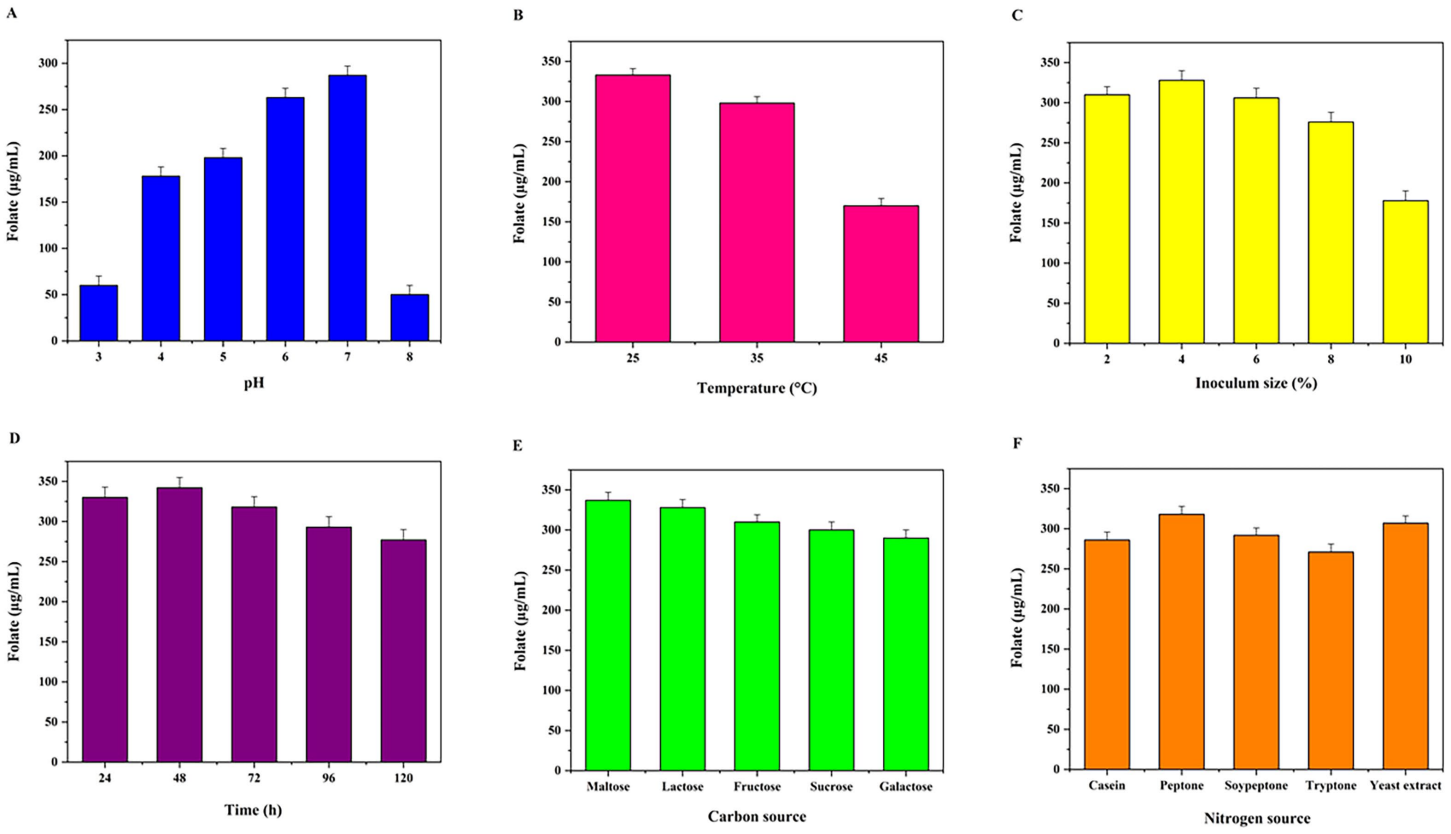
Preliminary screening of media components by OFAT analysis **(A)** Effect of various pH (3, 4, 5, 6, 7, and 8); **(B)** Effect of various temperatures (25, 35, and 45 °C); **(C)** Effect of different inoculum sizes (2, 4, 6, 8, and 10%); **(D)** Effect of various incubation times (24 h, 48 h, 72 h, 96 h, and 120 h); **(E)** Effect of various carbon sources (maltose, lactose, fructose, sucrose, and galactose); and **(F)** Effect of different nitrogen sources (casein, peptone, soy peptone, tryptone, and yeast extract) on folate production by *L. plantarum* MGKMVIT11.

Among the carbon sources evaluated, maltose proved to be the most effective for folate biosynthesis of the strain, yielding a maximum concentration of 337.27 μg/mL. This was closely followed by lactose, which supported a production level of 329.09 μg/mL. When the medium was supplemented with fructose or sucrose, folate concentrations reached 310 μg/mL and 300.61 μg/mL, respectively. In contrast, galactose was the least efficient substrate tested, resulting in the lowest output of 289.8 μg/mL. Figure 7 represents the preliminary screening of media components by OFAT analysis.

#### 6.9.2 Nitrogen sources

In the current study, peptone was identified as the most effective nitrogen source for folate biosynthesis. Using this substrate, the strain achieved a yield of 319.82 μg/mL. This was followed by yeast extract, which served as the second most efficient nitrogen source with a folate output of 307.84 μg/mL. Additionally, the inclusion of soy peptone in the production medium resulted in a folate concentration of 292.63 μg/mL. The production medium supplemented with casein and tryptone produced moderate levels of folate, at 285.51 μg/mL and 271.61 μg/mL, respectively.

#### 6.9.3 Temperature

Incubation temperature significantly influenced both biomass accumulation and folate yield of the strain. The peak folate concentration of 345.45 μg/mL was achieved at an optimal temperature of 25 °C. Although the strain maintained robust growth at higher temperatures of 35 °C and 45 °C, the corresponding folate levels decreased to 320.45 μg/mL and 170.71 μg/mL, respectively.

#### 6.9.4 pH

The pH significantly influenced the metabolic activity of the strain. Experimental data indicated that near-neutral conditions (pH 6 and 7) were optimal for both proliferation and biosynthesis, yielding folate concentrations of 263.55 μg/mL and 287.04 μg/mL, respectively. In contrast, extreme pH values—specifically pH 3 and 8—markedly inhibited these processes, with production dropping to 60.12 μg/mL and 49.90 μg/mL. Moderate yields were recorded at pH 4 (179.04 μg/mL) and pH 5 (198.06 μg/mL), confirming that a pH range of 6.0–7.0 is most conducive to folate recovery.

#### 6.9.5 Inoculum size

The effect of inoculum density on folate recovery was evaluated, revealing that a 4% concentration favored the highest output (329.47 μg/mL). While moderate yields (ranging from 276.29 to 311.31 μg/mL) were observed with 2, 6, and 8% inoculum, a further increase to 10% led to a substantial reduction in folate levels (178.45 μg/mL). These results suggest that excessive inoculum size may negatively impact the production rate.

#### 6.9.6 Incubation period

The incubation period of the folate-producing strain showed that 48 h was the optimum incubation period, yielding 341.59 μg/mL of folate. A moderate folate production of 329.61 μg/mL was observed at 24 h. When the incubation period extended to the death phase at 72, 96, and 120 h, the growth and yield were found to be 317.49, 292.59, and 276.88 μg/mL, respectively.

### 6.10 Plackett–Burman design

#### 6.10.1 Evaluation of factors affecting folate production

To identify the key determinants of folate biosynthesis, a PB design was implemented to evaluate eleven distinct nutritional and environmental variables. The experimental outcomes, summarized as mean folate concentrations (μg/mL) in Table 4, exhibited significant fluctuations across the different runs. Furthermore, the magnitude of the main effect for each parameter was determined to quantify its relative influence on production levels. Regression coefficient analysis of the eleven variables, including maltose, lactose, peptone, and ferrous sulfate, demonstrated beneficial effects on folate production. In contrast, ammonium sulfate, magnesium sulfate, NaCl, manganese sulfate, glucose, yeast extract, and KH2PO4 showed negative effects. The statistical significance of the model was evaluated using ANOVA, with the resulting data compiled in Table 5. The obtained results indicated a notable correlation. The fitted model, with R^2^ = 0.98962, accounts for variation in folate concentration. A strong association between the experimental and predicted folate concentrations was evidenced by a multiple R value of 0.991, demonstrating the high predictive accuracy of the model.

**Table 4:** Plackett–Burman design model for folate production by *L. plantarum* MGKMVIT11 and the observed design response.

S. no	Glucose (g/L)	Maltose (g/L)	Lactose (g/L)	Peptone (g/L)	Yeast extract (g/L)	(NH₄)₂SO₄ (g/L)	KH_2_PO_4_ (g/L)	NaCl (g/L)	FeSO_4_ (g/L)	MgSO_4_ (g/L)	MnSO₄ (g/L)	Folate concentration (μg/mL)
1)	10	30	10	10	10	2	1	0.01	0.01	0.1	0.025	289.0392157
2)	10	30	10	5	10	1	2	0.02	0.02	0.05	0.025	303.3529412
3)	30	10	10	10	5	2	2	0.02	0.01	0.05	0.025	283.3529412
4)	10	10	10	10	5	1	2	0.01	0.02	0.1	0.05	295.9019608
5)	30	30	10	5	5	2	1	0.01	0.02	0.05	0.05	290.8039216
6)	30	30	30	10	10	2	2	0.02	0.02	0.1	0.05	285.3137255
7)	30	10	10	5	10	1	1	0.02	0.01	0.1	0.05	288.6470588
8)	10	10	30	5	5	2	1	0.02	0.02	0.1	0.025	277.6666667
9)	30	10	30	10	10	1	1	0.01	0.02	0.05	0.025	301.7843137
10)	30	30	30	5	5	1	2	0.01	0.01	0.1	0.025	301.7843137
11)	10	10	30	5	10	2	2	0.01	0.01	0.05	0.05	283.745098
12)	10	30	30	10	5	1	1	0.02	0.01	0.05	0.05	307.0784314

**Table 5:** ANOVA of the experimental model of PB analysis.

Source	Degree of freedom	Sum of squares	Mean square	F ratio	Prob>F
Model	5	965.65424	193.131	114.4557	<0.0001*
Error	6	10.12431	1.687		
C. Total	11	975.77855			

*significant at *p* ≤0.0001.

According to the statistical analysis in Table 6, maltose, ammonium sulfate, and magnesium sulfate were identified as the critical determinants of folate biosynthesis, exhibiting a confidence level exceeding 90%. Consequently, less significant variables were excluded from subsequent optimization trials; these were maintained at either their low (−1) or high (+1) levels, depending on their individual influence on the output. Tables 5, 6 depict the ANOVA and effect tests of the PB analysis. Since the results suggested that, out of the eleven variables examined, ammonium sulfate, maltose, and magnesium sulfate significantly contributed to production levels, these variables were identified as critical for subsequent RSM via the CCD framework.

**Table 6:** Effect tests of PB analysis.

Source	Nparm	DF	Sum of Squares	F Ratio	Prob>F
Ammonium sulfate	1	1	654.56875	387.9190	<0.0001*
Maltose	1	1	178.44419	105.7519	<0.0001*
Magnesium sulfate	1	1	84.08305	49.8304	0.0004*
Sodium chloride	1	1	25.95156	15.3797	0.0078*
Peptone	1	1	22.60669	13.3975	0.0106*

Y predicted RMSE = 1.299, RSq = 0.98962, and *p value = <0.0001.

**Figure 8:**
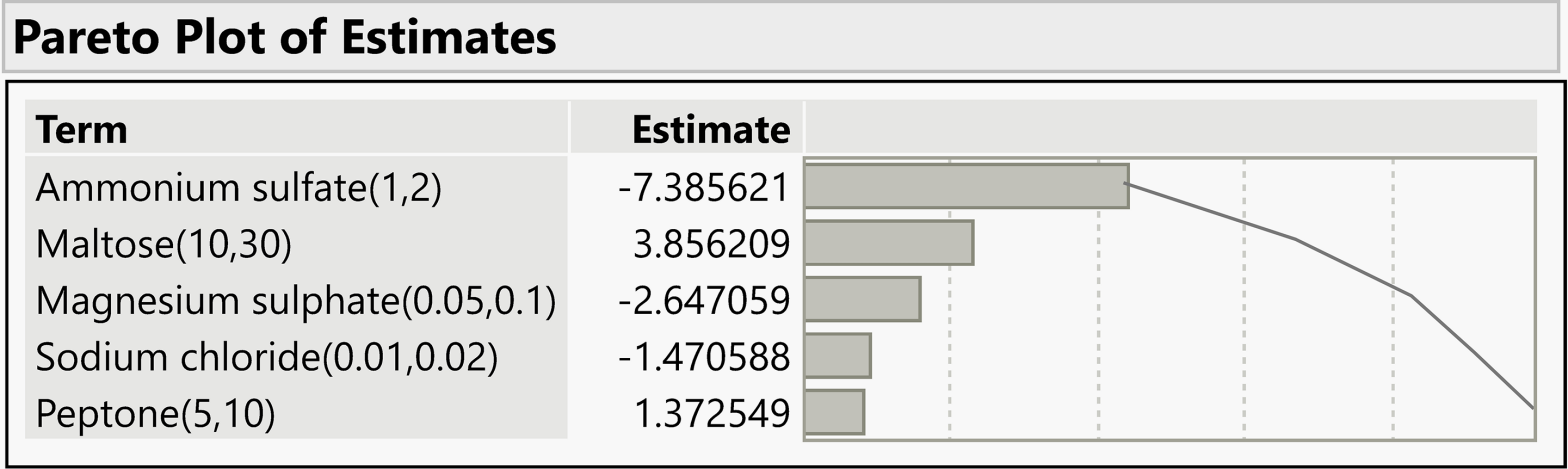
Pareto plot of estimates of PB analysis.

The Pareto chart (Figure 8) illustrates the main effects of individual variables on folate production. Analysis of the PB design identified ammonium sulfate, maltose, magnesium sulfate, sodium chloride, and peptone as statistically significant factors (*p* < 0.05). Among these, ammonium sulfate, maltose, and magnesium sulfate exhibited the highest effect estimates, contributing most substantially to the response variability. Consequently, these three variables were selected as critical factors for further optimization through CCD. To minimize model complexity and the total number of experimental runs, sodium chloride and peptone were maintained at their near-optimal levels in subsequent stages. Conversely, K_2_HPO_4_ and ferrous sulfate showed no significant effect on folate production. The model’s reliability was confirmed by a high coefficient of determination (R^2^ = 0.98962), a significant *p*-value (*p* < 0.0001), and a root mean square error (RMSE) of 1.299, indicating an excellent fit between the experimental and predicted data.

### 6.11 Statistical design-driven optimization of folate production

During preliminary optimization using the OFAT approach, the incubation period of 48 h resulted in the highest folate production (341.59 μg/mL) when evaluated as an independent variable. However, further multivariate optimization using CCD demonstrated that interactions among ammonium sulfate, maltose, magnesium sulfate, and incubation time significantly influenced folate biosynthesis, shifting the optimal incubation period to 24 h under statistically optimized conditions.

Following a 24 h fermentation period, the strain yielded 222.76 μg/mL folate in the non-optimized MRS broth. However, under optimized parameters (run 20), the biosynthetic output increased to a maximum of 364.35 μg/mL folate, with a corresponding biomass density (OD_600_) of 1.422. Statistical evaluation identified the linear model as the most appropriate fit for the data, characterized by the highest *F*-value. The robustness of the regression model was further validated by F-test analysis, which revealed a highly significant correlation with a negligible probability of error (*P* > *F* = 0.0010).

By adjusting the parameters, Table 7 presents the ammonium sulfate (A), maltose (B), and magnesium sulfate (C) at different concentrations; the CCD statistical design was used to enhance folate synthesis. According to the findings, Table 8 depicts responses 1 (folate μg/mL) and 2 (Y predicted values) that were achieved. ANOVA was performed to evaluate the concordance between the predicted and experimental outcomes, thereby validating the statistical reliability of the polynomial equation. Subsequently, a CCD incorporating six central points was employed to optimize the critical process parameters. Response 1, the folate yield, was investigated. Tables 9, 10 illustrate the ANOVA of the multiple regression and experimental analysis models using CCD.

**Table 7:** The experimental design in RSM includes independent variables and their corresponding levels.

Independent variable	Unit	Coded levels				
		-α	-1	0	+1	+α
Ammonium sulfate	g/L	0.6591035847	1	1.5	2	2.3408964153
Maltose	g/L	3.1820716949	10	20	30	36.817928305
Magnesium sulfate	g/L	0.0329551792	0.075	0.05	0.1	0.1170448208

**Table 8:** Optimization of folate production medium using central composite design and response analysis.

S. no	Ammonium sulfate (g/L)	Maltose (g/L)	Magnesium sulfate (g/L)	Folate concentration (μg/mL)	
				Experimental	Predicted
1)	0.6591035847	20	0.075	272.5882353	260.16497247
2)	2	30	0.1	262.0588235	275.34781196
3)	1.5	20	0.075	274.3529412	269.32581558
4)	2	10	0.1	257.1372549	263.94434356
5)	2.3408964153	20	0.075	224.5490196	227.9228612
6)	1.5	20	0.075	265.4117647	269.32581558
7)	1.5	20	0.075	264	269.32581558
8)	1	30	0.05	282.7843137	299.87360685
9)	1.5	20	0.0329551792	340.0980392	341.41222392
10)	1.5	20	0.1170448208	364.3529412	370.98933525
11)	1.5	3.1820716949	0.075	225.4509804	227.6146085
12)	1.5	20	0.075	265.5686275	269.32581558
13)	1.5	36.817928305	0.075	280.5882353	285.37518597
14)	2	30	0.05	277.6078431	280.01605829
15)	1.5	20	0.075	265.5686275	269.32581558
16)	2	10	0.05	285.745098	288.61258989
17)	1	10	0.1	280.9411765	282.42934312
18)	1.5	20	0.075	260.4901961	269.32581558
19)	1	10	0.05	241.9803922	242.58778555
20)	1	30	0.1	357.6862745	359.71516442

**Table 9:** ANOVA of the multiple regression model using CCD.

Source	Nparm	DF	Sum of squares	F ratio	Prob > F
A – Ammonium sulfate	1	1	1913.026	57.9912	<0.0001*
B – Maltose	1	1	3139.449	95.1688	<0.0001*
C – Magnesium sulfate	1	1	894.036	27.1017	0.0004*
AB	1	1	1823.014	55.2626	<0.0001*
AC	1	1	3121.275	94.6179	<0.0001*
BC	1	1	300.125	9.0979	0.0130*
A^2^	1	1	743.250	22.5308	0.0008*
B^2^	1	1	453.207	13.7384	0.0041*
C^2^	1	1	12513.147	379.3217	<0.0001*

Y predicted RMSE = 5.7435, RSq = 0.98749 and *p value = <0.0001.

**Table 10:** ANOVA of experimental analysis of CCD.

Source	Degree of freedom	Sum of squares	Mean square	F ratio	Prob>F
Model	9	26043.221	2893.69	87.7189	<0.0001*
Error	10	329.882	32.99		
C. Total	19	26373.103			

*significant at *p* ≤ 0.0001.

In this study, the linear coefficient for ammonium sulfate (*p* < 0.0001), maltose (*p* < 0.0001), and magnesium sulfate (*p* < 0.0004) emerged as highly significant model terms, exerting a profound influence on folate biosynthesis. As detailed in Table 11, the “lack of fit” was found to be non-significant (*F*-value is 2.1481), confirming that the experimental data align well with the proposed model. The adequacy of the developed regression model was evaluated using the coefficient of determination (R^2^), adjusted R^2^, and predicted R^2^ values. The model showed a high coefficient of determination (R^2^ = 0.98749), indicating that 98.749% of the variability in the response variable is explained by the model. The adjusted R^2^ value (0.976234) was also in good agreement with the R^2^ value, confirming the significance of the model. The predicted R^2^ value (0.99996), calculated using the PRESS statistic, further demonstrated the excellent predictive capability of the model. The close agreement among these statistical indicators confirms the reliability and adequacy of the developed regression model.

**Table 11:** Lack of fit.

Source	Degree of freedom	Sum of squares	Mean square	F ratio	Prob>F
Lack of fit	5	225.09603	45.0192	2.1481	0.2106
Pure Error	5	104.78617	20.9572		
Total Error	10	329.88220			

Max RSq = 0.9960.

**Figure 9:**
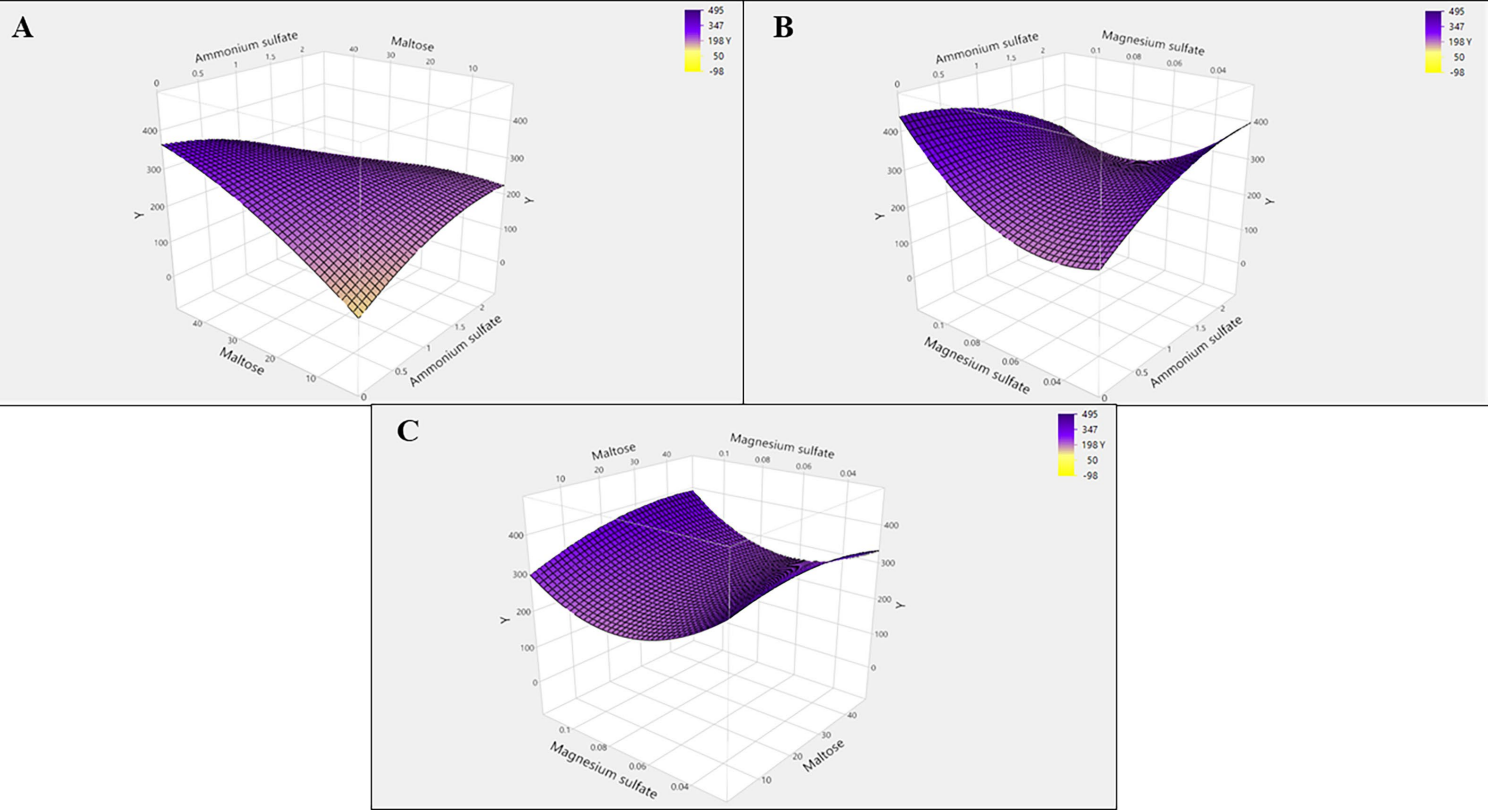
3D response surface plots for the interaction between independent variables on folate yield: **(A)** Ammonium sulfate and maltose, **(B)** ammonium sulfate and magnesium sulfate, and **(C)** maltose and magnesium sulfate.

Based on the ANOVA table, which exhibits three linear (A, B, C), three quadratics (A^2^, B^2^, C^2^), three interactions (AB, AC, BC), and one block term, the linear effect of ammonium sulfate and maltose is more pronounced than their quadratic effect. The findings corroborate the contour and response surface plots illustrated in Figure 9. The 3D response surface was based on the function of the concentrations of the two factors to establish the optimum level of each condition for folate yield maximization.

Figure 9A depicts the interaction between ammonium sulfate and maltose, while Figure 9B illustrates the interaction between ammonium sulfate and magnesium sulfate. Figure 9C represents the interaction between maltose and magnesium sulfate. The response surface curves exhibited a saddle-shaped profile, reflecting a clear interaction between the independent variables in the experimental model. The results showed that the highest production of folate occurs at the highest concentration of maltose and the mid-concentrations of ammonium sulfate and magnesium sulfate.

### 6.12 Antioxidant activity

**Figure 10:**
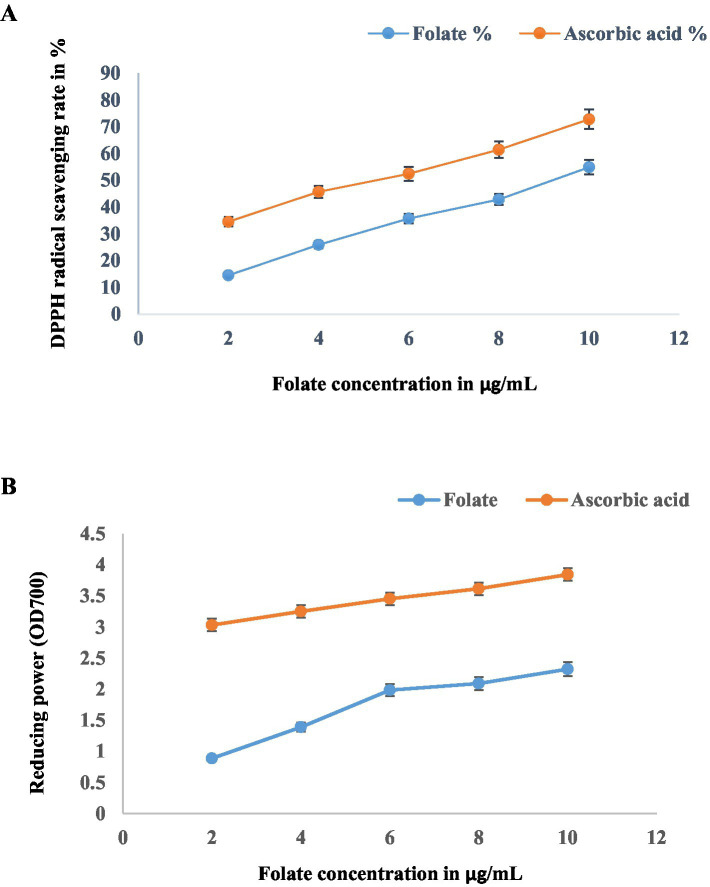
Antioxidant activity of folate on **(A)** DPPH radical and **(B)** reducing power, with ascorbic acid as a positive control; data shown as mean ± SD.

The antioxidant potential of folate, characterized by DPPH radical scavenging and reducing power assays, revealed a concentration-dependent efficacy (Figures 10A,B). While folate attained maximum values of 54.83 ± 0.72% (DPPH) and 2.322 ± 0.14 (reducing power) at 10 μg/mL, its overall performance indicates a moderate antioxidant potential in comparison to the ascorbic acid reference standard.

### 6.13 Anti-inflammatory effects of folate

#### 6.13.1 Evaluation of cytotoxicity

**Figure 11:**
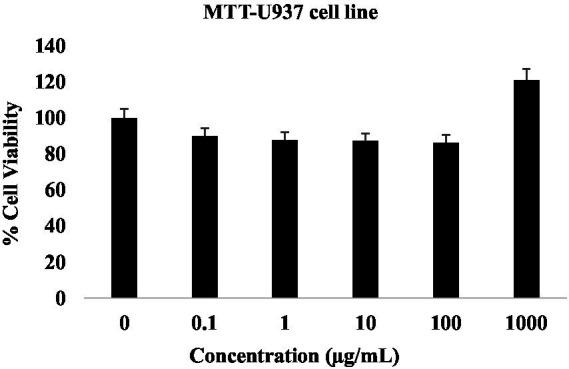
Effects of folate on cell viability of U937 cell lines.

Figure 11 shows the cytotoxicity of folate toward U937 cells evaluated using the MTT assay across a concentration range (0, 0.1, 1, 10, 100, and 1,000 μg/mL). Cell viability remained above 85% at concentrations up to 100 μg/mL, indicating minimal cytotoxic effects within this range. Notably, an increase in metabolic activity was observed at the highest tested concentration (1,000 μg/mL), where cell viability exceeded 120% relative to the untreated control group. To assess possible interference of the test preparation with the MTT assay, a blank control containing medium and MTT reagent without cells was included. The blank control showed negligible absorbance (average OD = 0.034), confirming the absence of direct interaction between the test preparation and the assay reagents. Therefore, the elevated viability values observed at higher concentrations likely reflect enhanced cellular metabolic activity rather than assay interference. Based on these findings, the IC_50_ value was determined to be > 1,000 μg/mL, indicating that folate is well tolerated by U937 cells and exhibits no detectable cytotoxicity within the evaluated concentration range.

#### 6.13.2 Cytokine gene expression analysis of IL-6, TNF-*α*, and IL-10 by qRT-PCR

**Figure 12:**
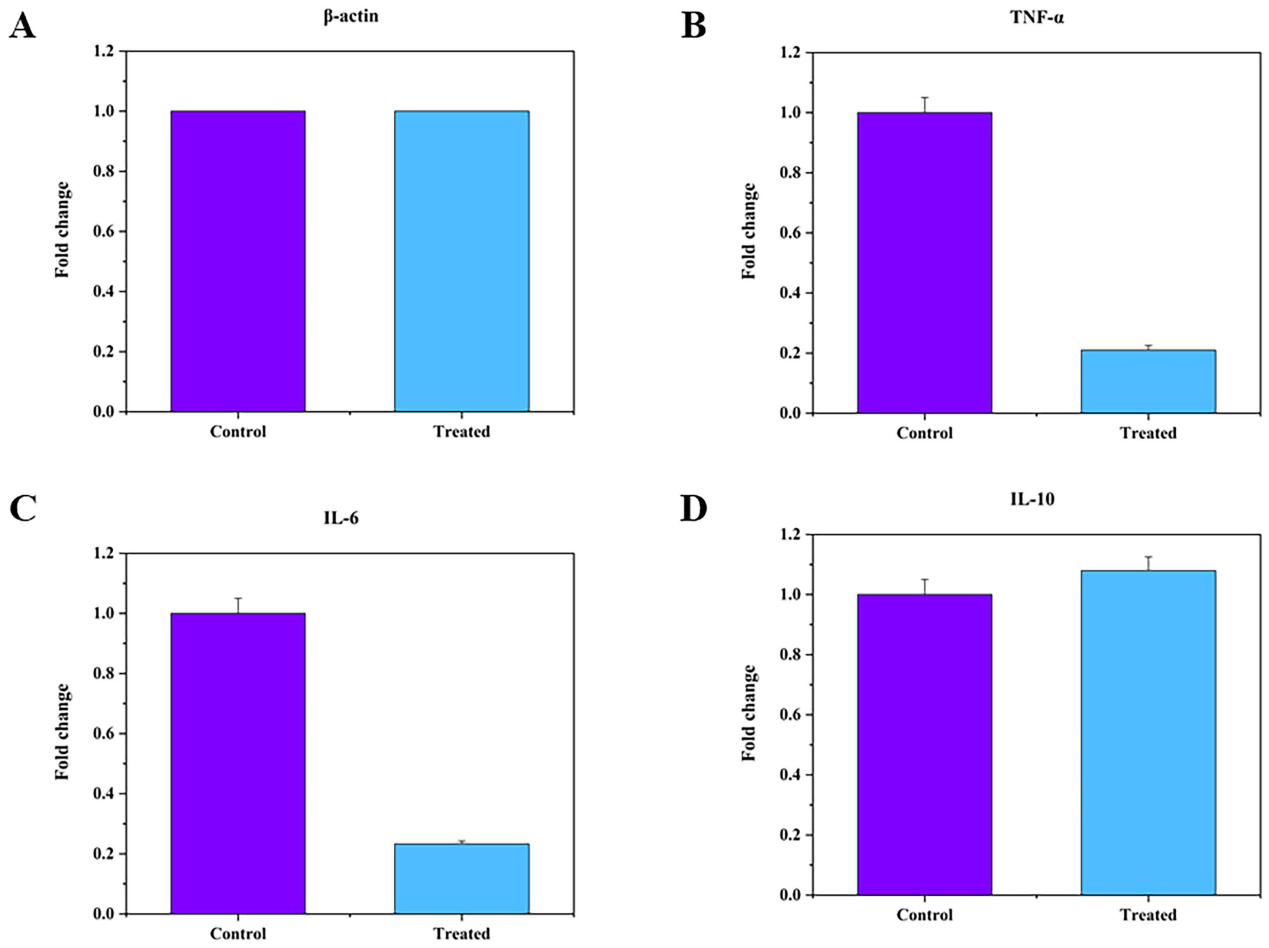
Effects of folate on the release of **(A)** *β*-actin (housekeeping gene), **(B)** TNF-*α*, **(C)** IL-6, and **(D)** IL-10 in stimulated U937 cells. Cells were conditioned with folate for 24 h, with cells seeded in the medium serving as the control.

In this study, the effect of folate on inflammatory cytokine gene expression (IL-6, TNF-α, and IL-10) was evaluated in LPS-stimulated U937 cells using quantitative real-time PCR. As shown in Figure 12, treatment with folate for 24 h significantly downregulated the expression of the pro-inflammatory cytokines TNF-α and IL-6 compared with the LPS-stimulated control group. In contrast, a slight increase in IL-10 expression was observed after folate treatment. Relative mRNA expression levels were calculated using the 2^(-ΔΔCt) method, and normalized to *β*-actin. The expression profiles (Figure 12) represent the mean ± SEM from independent experiments performed in triplicate.

### 6.14 Cytotoxic effects of the drugs on the HCT-116 cells

#### 6.14.1 Dose–response relationship between the drugs and HCT-116 cells

**Figure 13:**
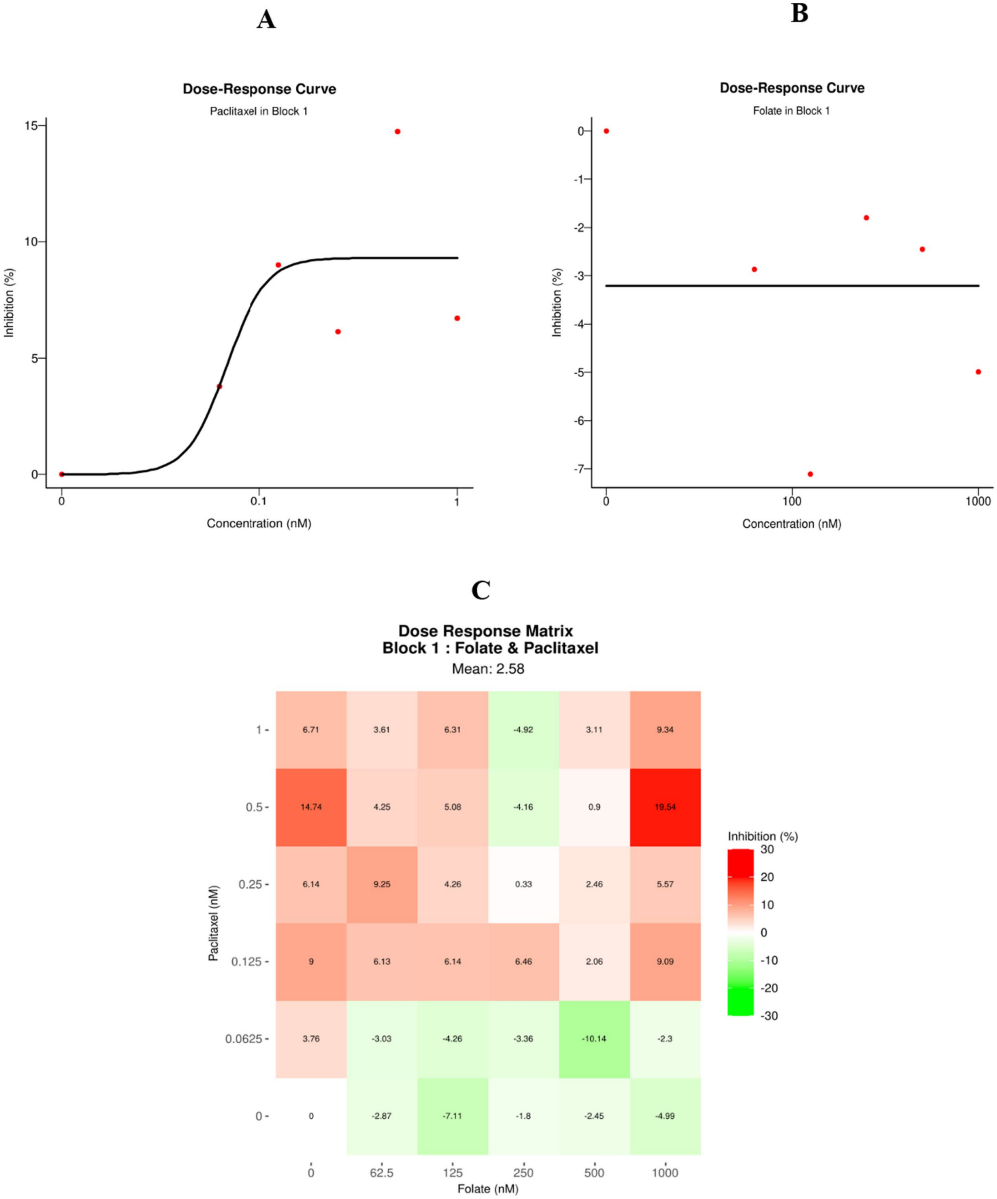
Cytotoxic effects of paclitaxel and folate on HCT-116 cells. **(A,B)** Dose–response curves for paclitaxel and folate monotherapies, respectively. **(C)** Combined dose–response profile of the two drugs.

From the analysis of the mono-therapeutic dose–response curves, Figures 13A,B revealed IC50 values of 0.07 nM for paclitaxel and 1,000 nM for folate, indicating that paclitaxel exhibits markedly higher potency than folate within the tested concentration range in HCT-116 cells. The evaluation of the combination treatment using the ZIP synergy model yielded a global mean synergy score of −1.37 (Figure 13C), indicating an overall additive interaction between paclitaxel and folate under the examined conditions. The mean inhibition across the tested concentration matrix was 2.58%. The highest synergy score (7.07) was observed at the concentration combination of 0.5 nM paclitaxel and 1,000 nM folate, corresponding to approximately a 19.54% reduction in cell viability relative to the untreated control.

#### 6.14.2 Degree of synergism between paclitaxel and folate

**Figure 14:**
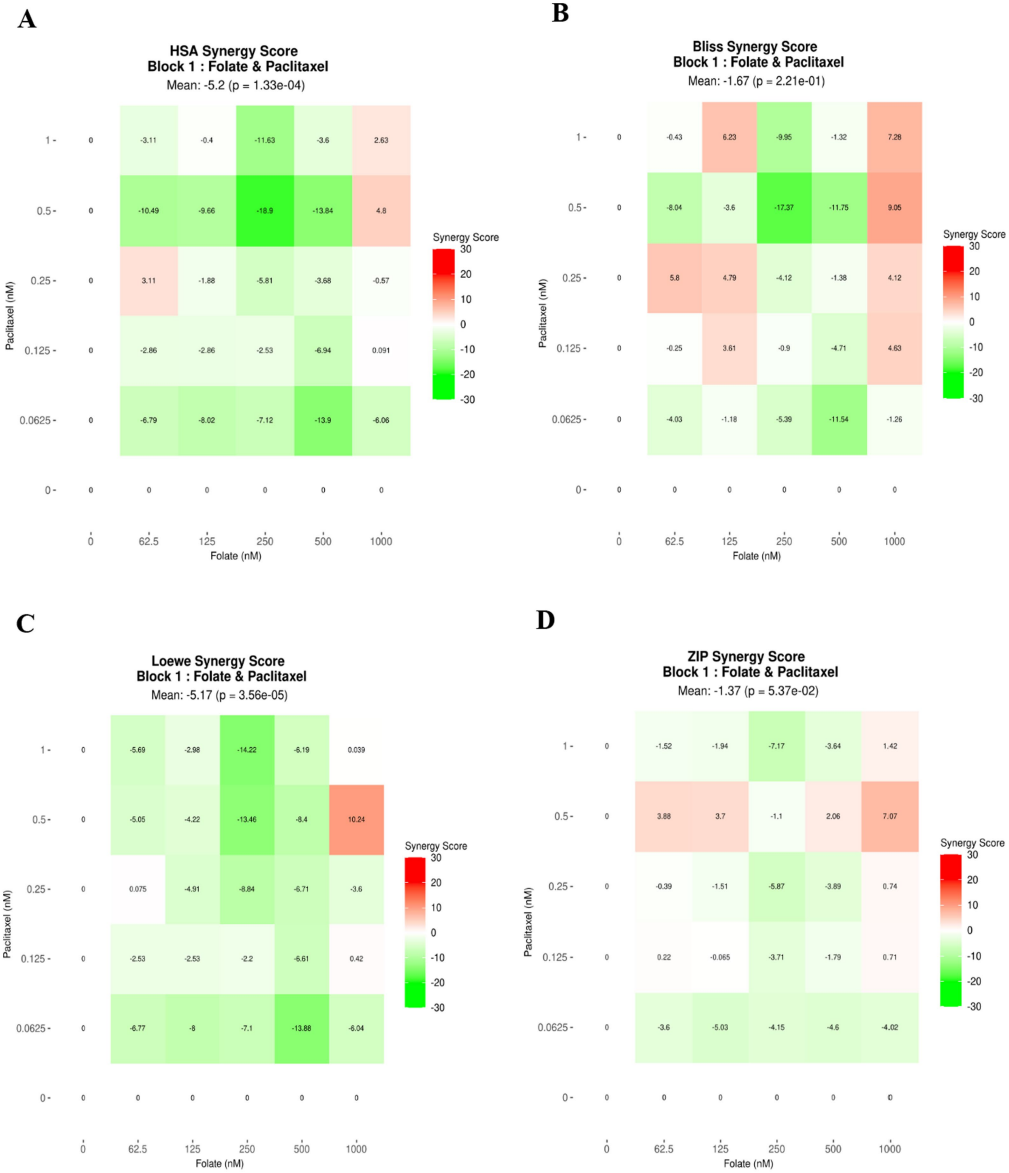
Synergistic interactions between paclitaxel and folate are illustrated as heatmaps generated from multiple computational models. Panels **A–D** correspond to the ZIP, Loewe, HSA, and Bliss frameworks, where red regions indicate positive energy scores and green regions denote antagonistic effects.

Table 12 and Figures 14A–D present the synergistic interactions between paclitaxel and folate, illustrated through four distinct synergy heatmaps derived from the ZIP, Loewe, HSA, and Bliss models. In these maps, red regions denote positive interaction scores, whereas green regions indicate antagonistic effects. The global mean synergy scores obtained from the ZIP (−1.37), Loewe (−5.17), HSA (−5.2), and Bliss (−1.67) models fall within the range of −10 to +10, confirming that the interaction between paclitaxel and folate in HCT-116 cells is predominantly additive rather than synergistic. These findings collectively support a concentration-dependent but overall additive interaction profile across the tested dose matrix.

**Table 12:** Synergy score summary.

Block ID	ZIP	Loewe	HSA	Bliss
1	−1.37	−5.17	−5.2	−1.67

As shown in Table 13, the individual sensitivity scores were −1.92 for folate and 6.35 for paclitaxel, whereas the combined treatment yielded a CSS value of 2.22. Since the combination sensitivity score did not exceed that of paclitaxel alone, the results further indicate that the combination does not support a synergistic cytotoxic interaction under the tested experimental conditions.

**Table 13:** Combination sensitivity table.

Block ID	Drug _1_	Drug_2_	IC50 _1_	IC50 _2_	RI_1_	RI_2_	CSS_1_IC50_2_	CSS_2_IC50_1_	CSS
1	Folate	Paclitaxel	1,000	0.07	−3.8	8.58	−1.92	6.35	2.22

## 7 Discussion

The present research focused on optimizing folate production using a potent *L. plantarum* MGKMVIT11 strain isolated from fermented *Sechium edule* (chayote), a non-dairy source. Our findings demonstrate that when inoculated in MRS medium, this strain produces 222.7647 μg/mL of folate. According to previous reports, the strain *Lactobacillus delbrueckii* KH1(Khedr et al., 2023) from a milk sample and *Lactobacillus delbrueckii* subsp. *bulgaricus* 25 (Hosseini et al., 2024) from traditional Iranian dairy products produced 100 μg/mL and 133.23 μg/mL, respectively. In addition, folate synthesized from non-dairy products was reported by the strain of *L. plantarum* TXZ2-26 (Guo et al., 2024) isolated from Sichuan kimchi and *L. plantarum* ZFM55 (Zhang et al., 2024), sourced from infant feces, which produced 39.2 ± 4.22 μg/mL and 299.72 ± 28.81 ng/mL of folate. The capacity for folate bio-enrichment varies considerably among strains; for instance, *L. plantarum* A3 has been shown to produce 64 μg/100 mL in plant-based milk fermentation systems (Tu et al., 2025). Similarly, vegetable-based fermentation, such as purple cabbage juice, has demonstrated substrate-dependent folate enhancement by *Lactiplantibacillus plantarum subsp. plantarum*, where total folate content increased by 44% (from 90.41 ± 5.69 ng/mL to 130.41 ± 9.07 ng/mL) after 6 h of fermentation (Tan et al., 2025). In addition, fermentation of a cauliflower–white bean mixture with *L. plantarum* increased folate levels by approximately 32–60% (58.8 ± 2.0 μg/100 g) compared with the unfermented control (Thompson et al., 2020). As observed in prior reports, fermentation of *Moringa oleifera* leaves powder using folate-producing strains such as *Lactiplantibacillus* sp. LP1 and *Saccharomyces cerevisiae* significantly enhanced the folate content to 1940 and 2,115 μg/100 g dry matter (Du et al., 2023a). Further, cereal-based fermentation studies, such as injera preparation, have shown that *L. plantarum* P2R3FA achieved the highest average folate yield (49.9 μg/100 g) in 100% sorghum flour (Tamene et al., 2023). Collectively, these findings indicate that folate production is strongly influenced by both strain characteristics and fermentation substrate. In this context, the current study is particularly relevant as it introduces chayote peel as a novel substrate for *L. plantarum*-mediated biosynthesis.

Probiotic safety is paramount, requiring confirmation that they do not negatively impact consumer health. The absence of hemolytic activity in strains *L. plantarum –* FS43, FS44, and FS48 (Kouadio et al., 2024), isolated from the traditional fermented paste, was determined by the hemolytic assay on blood agar plates (where no halo or zone of lysis is observed). *L. plantarum* and two other *Pediococcus* species from the Tilapia gut were also reported to be non-hemolytic (Coulibaly et al., 2023). Similarly, our strain also exhibited gamma hemolysis, which is a crucial indicator of its safety for potential applications in food or as probiotics. According to the report by Li et al. (2019), the strain *L. plantarum* can tolerate a pH as low as 2.5, measured at an absorbance OD value of 620 nm, which is 1.436 ± 0.062. Since the pH value in our stomach varies during fasting, it ranges from 1–2 to 4–5 after a meal. Our strain showed a similar ability; the strain maintained a high survival rate (75.32 ± 0.95%) even when exposed to simulated gastric fluid with a pH of 2.5 for 4 h. Research reports that *Bifidobacterium lactis* Probio-M8 (Liu et al., 2020) exhibited significantly higher tolerance to 0.3% bile salts compared to the two control strains (*p* < 0.05). Our strain also shows tolerance (78.16 ± 2.53%) to 0.3% bile salts. Sohn et al. (2020) showed that 30.61% auto-aggregation of *L. plantarum* LB5 exhibited excellent aggregation within the culture, and *L. plantarum* LB5 also demonstrated co-aggregation ability with four pathogens, including *L. monocytogenes* ATCC 51776 and *S. aureus* ATCC 25923, which showed the highest values at 61.30 and 62.69%, respectively, while *Escherichia coli* O157: H7 ATCC 35150 and *E. coli* KCTC 2571 had notably lower values at 33.45 and 33.08%. Similarly, the present study exhibits both auto-aggregation (70.32 ± 0.95%) and co-aggregation (68.51 ± 2.88%) of our strain. Among the solvents tested, the isolate showed a preferential affinity for xylene (48.37 ± 4.0%), indicating moderate cell surface hydrophobicity. Isolates JULABB01 and JULABB16 (Amenu and Bacha, 2023), isolated from Ethiopian traditional fermented foods and beverages, displayed the highest adherence capacities at 106 and 97%, respectively. In our current study, *L. plantarum* MGKMVIT11 shows excellent cell surface hydrophobicity with strong adherence to xylene (48.37 ± 4.0%). In a previous study, *Lactobacillus plantarum* WLPL041 isolated from human breast milk demonstrated susceptibility in an antibiotic susceptibility test, with susceptibility towards a 30 μg disc of chloramphenicol (23.87 ± 0.24) and a 15 μg disc of erythromycin (23.84 ± 0.19), complete resistance toward a 5 μg disc of rifampicin (0.00 ± 0.05), and intermediate susceptibility toward a 10 μg disc of streptomycin (3.84 ± 0.43) and a 10 μg disc of gentamicin (4.07 ± 0.14) (Jiang et al., 2016). Similar to our present study, our strain exhibits a moderate susceptibility zone of inhibition toward a 30 μg disc of chloramphenicol (18.2 ± 0.27), a 15 μg disc of erythromycin (17 ± 0.42), and a 5 μg disc of rifampicin (18 ± 0.45). Conversely, the strain exhibited complete resistance to a 10 μg disc of ampicillin (0.00 ± 0.05) and a 10 μg disc of streptomycin (0.00 ± 0.02), with intermediate susceptibility toward a 10 μg disc of gentamicin (12.5 ± 0.22). According to research reports, *L. pentosus* v390 exhibits a zone of inhibition against pathogenic bacteria such as *Bacillus subtilis* (9.50 ± 0.20 mm), *Escherichia coli* (7.10 ± 0.13 mm), *Listeria monocytogenes* (9.00 ± 0.46 mm), *Salmonella enterica* serovar Typhimurium (7.40 ± 0.29 mm), *Shigella dysenteriae* (7.00 ± 0.24 mm), and *Staphylococcus aureus* (9.90 ± 0.35 mm) (Behbahani et al., 2019). In our present study, the highest inhibitory effect was observed against *Salmonella enterica*, with a zone of inhibition measuring 17.1 ± 0.22 mm, compared to the zone of inhibition of *Bacillus cereus* (13.4 ± 0.61 mm) and *Shigella flexneri* (10.5 ± 0.53 mm).

This study explores the multifaceted dimensions of folate augmentation. Given that the culture medium must provide all precursors necessary for folate synthesis by probiotic cultures, its composition plays a critical role in biosynthesis efficiency. To minimize production costs, the medium was formulated using economical yet effective carbon and nitrogen sources. Additionally, key fermentation parameters, including incubation time, pH, temperature, and inoculum size, were optimized to maximize folate yields. For large-scale industrial production, it is essential to improve medium quality and culture viability before inoculation. According to Muhamad Nor et al. (2010), *L. plantarum* I-UL4 achieved a peak folate yield of 36.19 mg/L when supplemented with lactose, compared to maltose and glucose as carbon sources. Our findings indicate that the specific strain showed the highest folate yield of 337.27 μg/mL and 329.09 μg/mL when maltose and lactose were used as carbon sources, respectively, leading to the selection of maltose and lactose for further PBD study. In a previously reported study, the highest folate yields were achieved with a 15% inoculum of kombucha culture, reaching 69.52 μg/mL in fermented broccoli (after 6 days) and 62.05 μg/mL in fermented spinach (after 3 days), respectively (Melanie et al., 2017). Another research reported that optimizing the fermentation of barley and millet by *L. plantarum* MTCC 1407 (Srivastava et al., 2021) using RSM revealed that the maximum folate concentration (30 μg/100 g) was achieved at an optimum temperature of 40 °C and pH 5, with a fermentation time of 38.86 h.

In the present study, the folate production rate increased inversely with the growth rate of the organisms, reaching its maximum during the stationary phase. Based on preliminary results, the optimal medium composition was established at (g/L): maltose, 20; peptone, 10; yeast extract, 5; Tween 80, 1; ammonium sulfate, 2; sodium acetate, 5; magnesium sulfate, 0.1; manganese sulfate, 0.05; and K_2_HPO_4_, 2; with the incubation temperature and pH set at 25 °C and 7, respectively. A maximum of 441.5882 μg/mL of folate was achieved using a specific strain cultured in optimized medium, and the detected folate levels increased by 1.98 times compared to the unoptimized medium, which had a folate concentration of 222.7647 μg/mL. Folate production by *Propionibacterium freudenreichii* reached 39.2 ± 0.7 μg 100 mL^−1^ following statistical optimization of the medium via a PB experimental design (Zahed et al., 2022). Comparatively, our isolate showed a 1106.7 to 1147.0-fold increase in folate yield compared to previously reported strains. According to Muhamad Nor et al. (2010), the result from CCD indicated that the optimal concentrations of lactose, meat extract, and PABA yielded a maximum folate production of 60.39 mg/L. The optimized supplementation of urea, yeast extract, and MgSO₄·7H₂O within a CCD significantly increased protocatechuic acid production (5.27 g/L) using *C. glutamicum* (Chung et al., 2025). The study was conducted by optimizing experimental parameters using CCD, and the production of REPS by the strain *L. plantarum* RO30 (Elmansy et al., 2023) obtained a maximum yield of 10.32 g/L under optimized conditions, which consisted of 40 g/L sucrose, 25 g/L beef extract, pH 5.5, and a conditioned temperature of 30 °C for 72 h. RSM was employed to determine the optimal extraction conditions for folate from *Moringa oleifera* leaf powder. Under these optimized conditions – specifically a pH 7.0 extraction buffer with a 1:24 (w/v) mass-to-volume ratio, a rat serum volume of 350 μL, and a conjugase digestion time of 2 h – the microbial assay revealed a folate yield of 1190.06 ± 31.52 μg/100 g DM (Du et al., 2023b). To our knowledge, this study represents the first report on investigating the optimization of folate extracted from *L. plantarum* MGKMVIT11, producing the maximum amount of folate without adding any precursors to the medium, enriched with ammonium sulfate, maltose, and magnesium sulfate, which produced 364.35 μg/mL of folate was produced by specific strain cultured in the optimized medium. This represents a 1.64-fold increase compared to the yield obtained in the unoptimized medium.

Specifically, research on fermentation optimization has demonstrated DPPH inhibition ranges between 52 and 68% under specific environmental conditions. Notably, folate produced by the strain demonstrated significant antioxidant potential, with a scavenging activity of 54.83 ± 0.72% at 10 μg/mL. In addition, the reducing power of our folate reached 2.322 ± 0.14, a result that aligns with the robust reductive potential seen in other microbial compounds, where Ferric Reducing Antioxidant Power (FRAP) peaks at 6.57 mmol Fe^2+^ Eq/g (Srivastava et al., 2024). This suggests that folate achieves a similar magnitude of antioxidant performance without the need for complex fermentation, highlighting its efficiency as a radical scavenger and its potential as a stable bioactive component in therapeutic applications. Furthermore, the moderate antioxidant activity observed in this study is consistent with other reports on metabolites from the *Lactobacillus* genus. Our results for DPPH and reducing power of 54.88% and 2.322 (at 10 μg/mL) fall within the range reported for the EPS derivatives LPB8-0 and LPB8-1, isolated from *L. pentosus* B8, which exhibited DPPH and hydroxyl radical scavenging activities between 47.9 and 72.5% at the same concentration (10 mg/mL) (Jiang et al., 2022). This comparison validates folate’s radical-scavenging potential as a biologically relevant antioxidant. In addition, the therapeutic significance of folate synthesized by *Lactiplantibacillus plantarum* MGKMVIT11 is attributable to its involvement in one-carbon metabolism, nucleotide biosynthesis, and methylation reactions, which are integral to maintaining cellular redox homeostasis and regulating inflammatory signaling pathways (Lyon et al., 2020). Restoration of folate availability has been associated with the normalization of homocysteine metabolism, enhancement of endothelial function, and modulation of cytokine expression, thereby providing a mechanistic rationale for the antioxidant and immunomodulatory effects observed in the present study (Ayoub, 2025; Mölzer et al., 2021). These findings highlight the biological relevance of microbial folate biosynthesis and its potential contribution to host therapeutic management. Nevertheless, while these mechanisms are supported by experimental evidence, further clinical investigations are required to establish whether comparable therapeutic outcomes can be consistently achieved in human populations.

Inflammation is a multifaceted biological response triggered by microbial pathogens or their associated antigens, with macrophages serving as central mediators in both the initiation and progression of this process. When activated by LPS, these cells drive the inflammatory cascade by secreting a diverse array of pro-inflammatory cytokines (Giovanna et al., 2025). In the current study, to determine the biocompatibility of the synthesized folate, an MTT cell viability assay was performed using the U937 human monocytic cell model. This assay serves as a critical diagnostic tool, offering essential insight into cellular respiratory efficiency, which is fundamentally correlated with metabolic health; a viability threshold exceeding 80% is established as the benchmark for biocompatibility, whereas values falling below this limit suggest potential cytotoxic effects according to the guidelines outlined in ISO Standard 10,993–5 of 2009 (Cichos et al., 2023). In the current study, to determine the biocompatibility of the synthesized folate, an MTT cell viability assay was performed using the U937 human monocytic cell model. This assay serves as a critical diagnostic tool, offering essential insight into cellular respiratory efficiency, which is fundamentally correlated with metabolic health; a viability threshold exceeding 80% is established as the benchmark for biocompatibility, whereas values falling below this limit suggest potential cytotoxic effects according to the guidelines outlined in ISO Standard 10,993–5 of 2009. However, our study exhibits that a 24-h treatment with the test drug effectively modulates this cytokine profile in LPS-stimulated U937 cells. Specifically, the data reveal a significant downregulation of the pro-inflammatory markers TNF-*α* and IL-6, both showing a fold change reduction of approximately 80% compared to the control. Conversely, the treatment stimulated a modest upregulation of IL-10 levels. The stability of the *β*-actin housekeeping gene confirms that these observed differences reflect treatment-dependent modulation of cytokine gene expression rather than variability in RNA input or amplification efficiency. Recently, qRT-PCR and ELISA studies have shown that pre-treatment with *L. fermentum* KGC1601 induces a similar upregulation of anti-inflammatory cytokines and downregulation of pro-inflammatory mediators in the RAW 264.7 cell line (Kim et al., 2022). Collectively, these results demonstrate that the folate derived from *L. plantarum* MGKMVIT11 possesses robust anti-inflammatory potential, successfully counteracting LPS-induced inflammatory response by rebalancing the macrophage-mediated cytokine output.

Moreover, our study demonstrates a clear drug synergy between Paclitaxel and folate, emphasizing the value of rigorous synergy analysis in therapeutic optimization. By applying reference models such as HSA, Bliss, Loewe, and ZIP, we were able to distinguish between synergistic and antagonistic interactions, validating the enhanced efficacy of this specific combination. This quantitative validation supports the rationale for dose reduction, which is crucial for minimizing toxicity and maximizing therapeutic benefit in the treatment of clinical colorectal cancer (Duarte and Vale, 2022). Unlike the FOLFOXIRI regimen, which showed antagonistic interactions at clinical doses and limited improvement through optimized combinations (Zoetemelk et al., 2020), previous research explored targeted polymer–drug conjugates with varying folic acid/PEG ratios in HT-29 cells, demonstrating fold-enhanced activity relative to folate receptor expression (Grigoletto et al., 2021). In contrast, our study revealed that direct co-administration of Paclitaxel and folate produced an overall additive interaction profile in HCT-116 cells. Unlike conjugate compounds, where spatial separation of the targeting moiety and cytotoxic drug is critical, our findings highlight that folate may contribute to localized concentration-dependent enhancement of Paclitaxel response at specific dose combinations, supporting its potential role in combination-based therapeutic strategies for colorectal cancer.

## 8 Conclusion

This study primarily aimed to isolate and optimize the process parameters required for increased folate production. Among 9 isolates, a gram-positive strain of *Lactobacillus plantarum* MGKMVIT11 demonstrated the highest capacity to synthesize folate, which was confirmed by preliminary screening techniques using FACM and BCP indicator dye. To our knowledge, this is the first report of a folate-producing strain isolated from the fermented fresh peel of *Sechium edule* (chayote) vegetable. This strain exhibits good probiotic properties, including tolerance to high acidity and bile salt conditions, as a gamma-hemolytic strain, and resistance to 7 antibiotic discs, as well as antibacterial activity against various pathogens. Furthermore, scaling up the production of the folate-producing strain in a fermenter using optimized media led to a significant increase in vitamin yield. The observed experimental value (364.35 μg/mL) closely aligned with the RSM model’s predicted value (370.99 μg/mL). Given its high productivity in a safe, cost-effective medium, *L. plantarum* MGKMVIT11 represents a viable candidate for industrial food applications. Our study also focused on the antioxidant property of folate, which shows moderate DPPH radical scavenging and reducing power. Furthermore, *in vitro* anti-inflammatory activity of folate in LPS-stimulated U937 cells revealed a significant suppression of proinflammatory cytokines IL-6 and TNF-*α*, accompanied by a marked elevation in the anti-inflammatory cytokine IL-10. Together, the cell line–specific dose combinations of Paclitaxel and folate demonstrate an overall additive interaction profile against HCT-116 cells, with localized concentration-dependent enhancement at selected dose pairs that may support further investigation to improve therapeutic outcomes beyond conventional drug regimens.

## Data Availability

The dataset presented in this study can be found in online repositories. The name of the repository and accession number can be found at: https://www.ncbi.nlm.nih.gov/genbank/, and OR018545.

## Footnotes

1
https://synergyfinder.org/#!/
