# Supplementary material for: Production optimization and in vitro evaluation of the biofunctional potential of folate from Lactiplantibacillus plantarum-MGKMVIT11
Source: Front Microbiol. 2026 May 15;17:1811272. doi: 10.3389/fmicb.2026.1811272 (PMC13219362; doi:10.3389/fmicb.2026.1811272)
Supplement: Supplementary file 1 [file Table_1.docx]

**Production Optimization and *In Vitro* Evaluation of the Biofunctional Potential of Folate from *Lactiplantibacillus plantarum*-MGKMVIT11**

G Megala and M Kavitha*

School of Bio Sciences and Technology, Vellore Institute of Technology, Vellore-632014,

Tamil Nadu, India

^*^Corresponding Author

E-mail: [mkavitha1972@gmail.com](mailto:mkavitha1972@gmail.com)

Tel.: +914162243091

ORCID: 0000-0002-1862-8037

**Supplementary Figure 1. Folic acid standard curve**
